# Effects of age on electrophysiological measures of cochlear synaptopathy in humans

**DOI:** 10.1016/j.heares.2020.108068

**Published:** 2020-10

**Authors:** Samuele Carcagno, Christopher J. Plack

**Affiliations:** a Department of Psychology, Lancaster University, Lancaster, LA1 4YF, United Kingdom; b Manchester Centre for Audiology and Deafness, University of Manchester, Manchester Academic Health Science Centre, M13 9PL, United Kingdom

**Keywords:** Cochlear synaptopathy, Presbycusis, Auditory brainstem response, Frequency following response

## Abstract

Age-related cochlear synaptopathy (CS) has been shown to occur in rodents with minimal noise exposure, and has been hypothesized to play a crucial role in age-related hearing declines in humans. Because CS affects mainly low-spontaneous rate auditory nerve fibers, differential electrophysiological measures such as the ratio of the amplitude of wave I of the auditory brainstem response (ABR) at high to low click levels (WI_H_/WI_L_), and the difference between frequency following response (FFR) levels to shallow and deep amplitude modulated tones (FFR_S_-FFR_D_), have been proposed as CS markers. However, age-related audiometric threshold shifts, particularly prominent at high frequencies, may confound the interpretation of these measures in cross-sectional studies of age-related CS. To address this issue, we measured WI_H_/WI_L_ and FFR_S_-FFR_D_ using highpass masking (HP) noise to eliminate the contribution of high-frequency cochlear regions to the responses in a cross-sectional sample of 102 subjects (34 young, 34 middle-aged, 34 older). WI_H_/WI_L_ in the presence of the HP noise did not decrease as a function of age. However, in the absence of HP noise, WI_H_/WI_L_ showed credible age-related decreases even after partialing out the effects of audiometric threshold shifts. No credible age-related decreases of FFR_S_-FFR_D_ were found. Overall, the results do not provide evidence of age-related CS in the low-frequency region where the responses were restricted by the HP noise, but are consistent with the presence of age-related CS in higher frequency regions.

## Highlights

• We used highpass masking to record ABRs and FFRs from low-frequency regions.
• Wave I ABRs with highpass masking are not consistent with low-frequency synaptopathy.
• Wave I ABRs without highpass masking are consistent with high-frequency synaptopathy.
• Highpass-masked FFRs do not provide evidence of low-frequency synaptopathy.

## 1 Introduction

Hearing loss is one of the most common chronic conditions in older adults (Lin et al., 2011). Besides a loss of sensitivity at high frequencies, age-related hearing declines include deficits in processing sounds at suprathreshold levels, and difficulties understanding speech in noise (Humes et al., 2012). While some of these declines may be due to dysfunction of the outer hair cells (OHCs), and of the inner hair cells (IHCs) in the cochlea (Schmiedt, 2010), there is increasing evidence of contributions from other age-related physiological changes at the level of the cochlea, and of the central auditory system (Caspary, Ling, Turner, Hughes, 2008, Ouda, Profant, Syka, 2015).

Cochlear synaptopathy (CS) has been hypothesized to play a major role in age-related hearing declines (Kujawa, Liberman, 2015, Liberman, Kujawa, 2017). CS has been widely documented in rodents as a result of acoustic trauma: Noise exposures titrated to cause only temporary threshold shifts, in the absence of permanent OHC damage, have been shown to result in a permanent loss of synapses between the IHCs and auditory nerve fibers (Kujawa and Liberman, 2009). This loss of afferent synapses is thought to affect mainly auditory nerve fibers with low and medium spontaneous firing rates (L/M-SR fibers) that are considered to be important for coding sounds at high levels. Direct evidence for a greater involvement of L/M-SR fibers in CS was obtained in guinea pigs by Furman et al. (2013). Indirect evidence comes from the observation that while CS does not affect neurophysiological responses at low stimulus levels, it leads to reductions of neurophysiological responses at supra-threshold stimulus levels, in particular of wave I of the auditory brainstem response (ABR), of the frequency following response (FFR) to high-frequency ( ~ 1-kHz) amplitude modulation (Shaheen et al., 2015), and of the middle ear muscle reflex (Valero, Hancock, Liberman, 2016, Valero, Hancock, Maison, Liberman, 2018).

Sergeyenko et al. (2013) observed age-related CS in CBA/CaJ mice raised in a quiet environment. They found that IHC synaptic counts, estimated from IHC ribbon survival, progressively declined across the lifespan. This loss of afferent synapses was mirrored by progressive reductions of wave I of the ABR at supra-threshold levels that occurred before significant changes in distortion product otoacoustic emissions (DPOAEs), which index OHC function, and in wave I ABR thresholds were observed. Noise exposure titrated to cause only temporary threshold shifts at a young age has been shown to accelerate age-related CS (Fernandez et al., 2015).

Parthasarathy and Kujawa (2018) also observed a progressive decline of IHC synaptic counts with age that preceded hair cell losses in CBA/CaJ mice. They measured FFRs to 1024 Hz amplitude modulated (AM) tones at several levels, and modulation depths (MDs). FFR amplitudes were generally reduced as a function of age across levels. As predicted by the CS model of Bharadwaj et al. (2014), FFR growth functions with level became progressively shallower as a function of age, but at equal sensation levels rather than at equal SPLs. FFR amplitude at a sensation level of 30 dB correlated with the degree of synaptic loss. However, in contrast to the prediction of the Bharadwaj et al. (2014) model, FFR growth functions with MD had similar shapes across the age range. Age-related declines of IHC ribbon synapses have also been observed in gerbils (Gleich et al., 2016).

Evidence consistent with age-related CS in humans comes from post-mortem studies of temporal bones. After synaptic disconnection the peripheral axons of spiral ganglion neurons (SGNs) degenerate, followed after a delay by the SGNs bodies. Post-mortem studies of human temporal bones have shown steady age-related declines of SGN peripheral axons (Wu et al., 2019), IHC synaptic ribbons (Viana et al., 2015), and SGN bodies (Makary et al., 2011), that precede or exceed hair cell loss. However, it is unclear whether the age-related degeneration of SGNs found in human temporal bones mainly affects L/M-SR fibers.

Two recent studies have sought to identify a neural correlate of age-related CS in humans by measuring wave I ABR amplitude as a function of stimulus level, which is expected to have a shallower growth rate as a result of CS affecting mainly L/M-SR fibers. Johannesen et al. (2019) measured wave I amplitudes at levels from 90 to 110 dB ppeSPL in a group of 94 participants ranging in age from 12 to 68 years. The growth of wave I amplitude with level decreased with age, and this effect was still present when wave I amplitude/level slopes were adjusted for the effect of audiometric thresholds at 12 kHz. This result suggests that the effect of age on ABR amplitude growth was not simply due to age-related increases in high-frequency audiometric thresholds.

Grose et al. (2019) measured wave I ABR amplitude at levels of 95, and 105 dB ppeSPL in a group of 10 young, and a group of 10 older listeners. They found that wave I amplitude growth with stimulus level was reduced in the older listener group. Although the older listener group had near-normal hearing up to 4 kHz, many had varying degrees of hearing loss above 4 kHz.

An important factor to consider when interpreting the results of these studies is that the cochlear regions contributing to wave I depend on stimulus level. The ER-3A (Etymotic Research Inc., Elk Grove, U.S.A.) earphones commonly used in these studies (e.g. Grose, Buss, Elmore, 2019, Johannesen, Buzo, Lopez-Poveda, 2019) have a low-pass frequency response with a spectral plateau from about 1.5 to 4 kHz. While at low stimulus levels basilar membrane excitation is restricted around cochlear sites with characteristic frequencies close to those of the stimulus, as the level increases the excitation spreads towards the base of the cochlea (i.e. towards cochlear places with higher characteristic frequencies) (Robles, Ruggero, 2001, Ruggero, Rich, Recio, Narayan, Robles, 1997). Moreover, the contributions of more basal sites tend to dominate ABR wave I (Don, Eggermont, 1978, Eggermont and Don, 1980) because traveling wave dispersion is lower at more basal cochlear sites, leading to more synchronized firing of neurons with high characteristic frequencies (Dau, 2003, Dau, Wegner, Mellert, Kollmeier, 2000). A recent study (Lee et al., 2019) also found that compound action potentials to high-level tone bursts recorded in non-human animals, which are often assumed to originate predominantly from the cochlear place at the characteristic frequency of the tone, can originate from cochlear regions far offset from the characteristic frequency. Responses to low and high level stimuli may thus differ not only for the types of neurons [L/M-SR or high spontaneous rate (H-SR)] contributing to them, but also for the cochlear regions contributing to them. A greater reduction of wave I amplitude to high compared to low level stimuli may thus reflect greater sensorineural deficits (possibly other than CS) in more basal cochlear regions compared to more apical cochlear regions, rather than deficits specifically affecting L/M-SR fibers. Therefore, a shallower growth of the ABR wave I amplitude/level function with age, while consistent with CS, does not provide compelling evidence in its favor. Comparing ABRs to high and low level stimuli obtained with highpass masking techniques that restrict the cochlear region from which the responses originate (Don, Eggermont, 1978, Eggermont and Don, 1980) would provide a more specific test of the CS hypothesis.

Restricting the cochlear region from which the ABRs originate would additionally make it easier to disentangle possible CS effects from the effects of age-related losses of hearing sensitivity, which are especially prominent at high frequencies. Dysfunction of the OHCs is expected to decrease mainly responses at low stimulus levels, producing *steeper* wave I amplitude/level functions (Verhulst, Jagadeesh, Mauermann, Ernst, 2016, Verhulst, Altoe, Vasilkov, 2018), rather than the shallower amplitude/level functions predicted by CS. However, this picture is likely to be more complex if one considers dysfunction of the IHCs as an additional source of loss of hearing sensitivity, because IHC dysfunction may reduce responses both at low and at high stimulus levels, and may give rise to shallow wave I amplitude/level functions (Heinz, 2015, Heinz, Young, 2004, Saremi, Stenfelt, 2013). It is possible to use audiometric thresholds as covariates to statistically partial out the effect of age-related losses of hearing sensitivity on ABRs, as done by Johannesen et al. (2019). However, one issue with this approach is that wide cochlear regions may contribute to neurophysiological responses: using thresholds at each audiometric frequency as covariates can lead to overfitting, while using average thresholds over wide frequency regions can be problematic if they do not contribute equally to the neurophysiological response. Restricting the cochlear regions from which the responses originate makes it easier to statistically control for possible audiometric confounds; moreover, these cochlear regions can be restricted to those low-frequency regions that are least affected by age-related hearing loss.

In the current study, ABRs and FFRs were recorded in the presence of a highpass masking noise that effectively restricted the cochlear sites where the responses originate to low-frequency regions (Don and Eggermont, 1978) with minimal differences in audiometric thresholds across the age range. Residual age-related audiometric threshold differences within this restricted cochlear region were also partialed out statistically. In addition, ABRs were also recorded in quiet, and average audiometric thresholds at low and high frequency regions were used as a covariates to minimize possible confounding effect of hearing loss.

On the basis of previous models of CS (Bharadwaj, Verhulst, Shaheen, Liberman, Shinn-Cunningham, 2014, Plack, Leger, Prendergast, Kluk, Guest, Munro, 2016) we used differential measures to separate the effects of CS from general age-related reductions of electrophysiological responses. In particular, we assessed the ABR wave I amplitude ratio between responses at high and low stimulus levels, and the FFR amplitude difference between AM tones with shallow and full MD. The predictions of CS models on differential measures are based on the assumption that CS affects mainly L/M-SR fibers (Bharadwaj, Verhulst, Shaheen, Liberman, Shinn-Cunningham, 2014, Grose, Buss, Elmore, 2019, Johannesen, Buzo, Lopez-Poveda, 2019, Plack, Leger, Prendergast, Kluk, Guest, Munro, 2016). This will be the working assumption on which the evidence for age-related CS in the current study will be assessed. However, in the Discussion, we will also consider the possibility that this assumption is, at least for age-related CS, incorrect. ABRs and FFRs were acquired for a large cohort (n=102) of participants across the age range (18–73). These electrophysiological recordings were part of a larger study on the same cohort of participants that included psychophysical measures of temporal coding, measures of speech perception in noise, and cognitive measures. This paper will present only the results of the electrophysiological tests, the results of the other tests, and their relations to the electrophysiological results will be presented in future papers.

## 2 Methods

### 2.1 Participants

A total of 170 participants from three age groups (young: 18–39, middle-aged: 40–59, older:  > 60 years old) were enrolled in the study. Sixty-eight participants either failed to meet the selection criteria outlined below, or withdrew from the study. Only the data of the 102 participants who completed the study will be presented. Selection criteria included audiometric thresholds for both ears below 20 dB HL at octave frequencies from 0.125 to 2 kHz, and below 40 dB HL at 4 kHz. No selection criteria were imposed for frequencies above 4 kHz. Participants with audiometric threshold asymmetries between the left and right ear larger than 20 dB at any frequency from 0.125 to 4 kHz were excluded from the study. Due to the use of an incorrect calibration table for the headphones used in the audiometric tests the actual cutoff thresholds differed by a few dBs with respect to the nominal cutoff thresholds listed above. Using the correct calibration table, five older, two middle-aged, and two young participants would not have passed the selection. However, these listeners had thresholds below 30.5 dB HL for audiometric frequencies up to 2 kHz, and below 37 dB HL at 4 kHz. Given that their thresholds were only slightly above the cutoff criteria, and given that audiometric thresholds were used as continuous covariates, the data of these listeners were included in the analyses. An otoscopic examination was performed prior to the beginning of the tests, and participants with earwax occlusions were excluded from the study.

Recruitment continued until 34 participants from each age group had completed the study. Within each age group 27 females, and seven males completed the study. Towards the end of the study recruitment was targeted to ensure that the proportion of females to males would be the same across the three age groups.

Participants were asked to report the number of years of musical practice (with a musical instrument or vocal) they had. They gave written informed consent for participation in the study, and received an hourly wage. All the experimental procedures were approved by the Lancaster University Research Ethics Committee.

### 2.2 Recording procedures

EEG responses were recorded using a Biosemi ActiveTwo (BioSemi B.V., Amsterdam, The Netherlands) system with a 16,384 Hz sampling rate. Gold-plated cup electrodes were placed on the forehead just below the hairline (high forehead; HF), on each mastoid, and on the neck at the level of the 7th cervical vertebra (C7). Gold-plated clip electrodes were attached to each earlobe. Gold foil tiptrodes were used to deliver the stimuli and provide additional electrodes in the ear canal. The common mode sense and driven right leg electrodes were place on the forehead. During the recording listeners reclined comfortably on a reclining chair in a double-walled IAC (IAC Acoustics, Winchester, UK) soundproof booth, and were asked to relax and refrain from extraneous body movements. The stimuli were generated digitally with a 32-bit resolution, and a 48-kHz sampling rate in Python (Python Software Foundation, Delaware, United States); they were sent to a 24-bit RME Hammerfall DSP multiface digital-to-analog converter (RME Intelligent Audio Solutions, Germany), and were played via mu-metal shielded Etymotic ER-3A insert earphones in rarefaction polarity. Triggers marking the start of a stimulus were sent to the Biosemi receiver from additional channels of the soundcard after being transformed to discrete pulses by a custom-built device. The EEG data were processed offline using custom scripts written in Julia (Bezanson et al., 2017).

### 2.3 ABR Stimuli

**Fig. 1:**
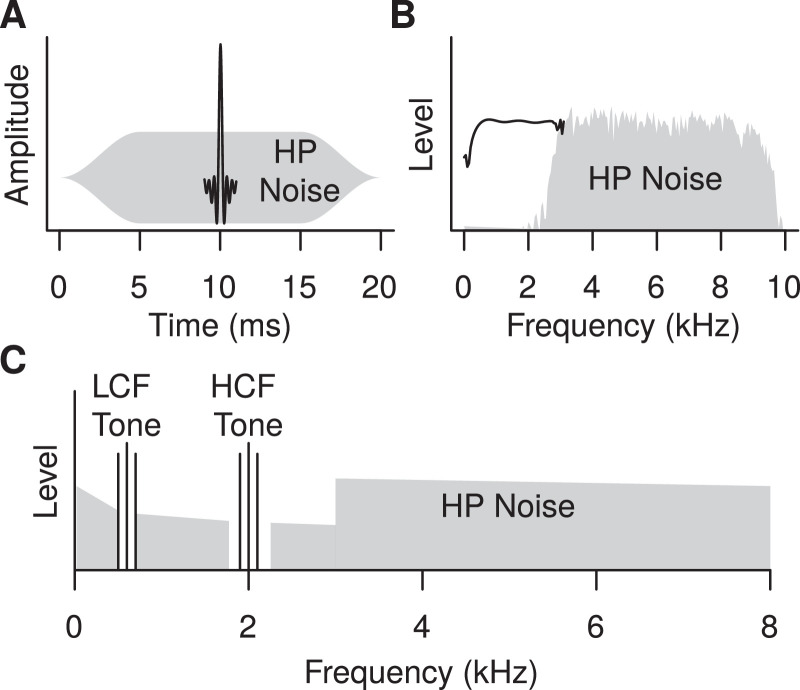
Schematic representation of the stimuli used in the study. **A.** Time domain, and **B.** frequency domain representations of the filtered click embedded in the highpass masking noise used for the ABR experiment. **C.** Frequency domain representation of the AM tones and HP masking noise used in the FFR experiment.

A 100-µs click was bandpass filtered between 0.35 and 3 kHz. Two milliseconds of the output sequence resulting from the convolution of the click and the filter centered at the peak of the click was used as the stimulus. The clicks were presented at levels of 105 and 80 dB ppeSPL. They were either presented in quiet, or were embedded in a 20-ms burst of highpass (HP) pink noise filtered between 3.5 and 8 kHz. Schematic time-, and frequency-domain representations of the click embedded in HP noise are shown in Figs. 1A, and 1B, respectively. To minimize the ABR to the onset of the noise, the noise was gated on and off with 5-ms raised-cosine ramps. Furthermore, the click onset time was drawn randomly from a uniform distribution between 5 and 13 ms after the onset of the noise on each stimulus presentation. Because the averaging was synchronized to the onset of the click, the resulting 8-ms jitter in the relative onset of the noise should have eliminated the contribution of the noise onset to the ABR average. The noise had a spectrum level of 65 and 40 dB SPL at 1 kHz, respectively for the 105, and 80 dB ppeSPL clicks. In pilot studies this noise level was found to completely mask the response to a click bandpass filtered between 0.35 and 8 kHz when the noise was bandpass filtered in the same frequency region.

Ten thousand stimuli were presented for each stimulus condition. To minimize the recording time the clicks were presented alternately to each ear. The stimuli were presented at a combined rate across the two ears of 14.1 stimuli per second (the silent interval between noise bursts was 50.9 ms). Monoaurally the stimulus presentation rate was of 7.05 stimuli per second.

### 2.4 ABR Processing

The continuous EEG recordings were bandpass filtered offline between 0.1 and 1.5 kHz using a 256-taps zero-phase-shift finite-impulse-response (FIR) filter. The data from the HF electrode were referenced to the ipsilateral earlobe (IERL), ipsilateral tiptrode (ITPR), and ipsilateral mastoid (IMST) electrodes to obtain three different montages that are commonly used in ABR studies (Hood, 2015, Møller, 2006). However, the data from the HF-IMST montage were not retained for subsequent analyses. The rationale for this choice is detailed in the Supplementary Materials (SM). The HF-ITPR and HF-IERL data were modeled jointly in the statistical analyses; however, for brevity, and because i) the results across the two montages were largely similar, ii) the HF-ITPR electrode may provide a larger and slightly more reliable wave I due to its proximity to the wave I generator (Bauch, Olsen, 1990, Prendergast, Tu, Guest, Millman, Kluk, Couth, Munro, Plack, 2018), only the HF-ITPR results will be shown and discussed in the main manuscript. The HF-IERL results are shown in the SM.

The triggers marking the onset of the stimuli in the EEG recordings were delayed by 0.9 ms to compensate for the acoustic delay introduced by the earphone tubes. The recordings were then segmented from -3 to 12 ms relative to click onset, and baseline corrected using a 3-ms baseline. The segments for each stimulus, montage, and ear were averaged using an iterative-weighted average (Riedel et al., 2001) for each participant. Responses from the left and right ears were then averaged together to increase the signal-to-noise ratio (SNR). Grand averages for each stimulus were computed across all participants.

ABR peaks and troughs for wave I and wave V were identified using a semi-automatic peak-picking procedure which is described in detail in the SM.

### 2.5 FFR Stimuli

FFRs were elicited by two simultaneous diotic AM tones, one with a low carrier frequency (CF) of 0.6 kHz (LCF tone), and one with a high carrier frequency of 2 kHz (HCF tone). A schematic representation of the stimuli is shown in Fig. 1C. The tones had a duration of 450 ms, including 10-ms raised-cosine on and off ramps. On each trial the low and high CF tones were always modulated at a different modulation frequency (MF) close to 100 Hz (MF1=93.3, MF2=102.2, MF3=111.1, MF4=120 Hz). There were four stimulus configurations resulting from the combination of the CF/MF employed: LCF_MF1_/HCF_MF3_, LCF_MF2_/HCF_MF4_, LCF_MF3_/HCF_MF1_, LCF_MF4_/HCF_MF2_. The two tones were both modulated either with 100%, or with 70% MD.

The AM tones were generated by sinusoidally amplitude modulating a 75 dB SPL pure tone. Therefore the level of the resulting AM tones was 76.76 dB SPL for the tone with 100% MD, and 75.95 dB for the tone with 70% MD. The tones were embedded in pink noise to reduce the contribution of H-SR fibers to the recorded FFRs. The pink noise had a spectrum level of 40 dB SPL at 100 Hz, and was presented in a frequency region from 20 to 3000 Hz, with notches two equivalent rectangular bandwidths (Glasberg and Moore, 1990) wide around the CFs so as to form three noise bands (20–515, 694–1773, and 2253–3000 Hz). A pink noise bandpass filtered from 3 to 8 kHz, with a spectrum level of 50 dB SPL at 4 kHz was presented with the AM tones to eliminate the contribution of high-frequency cochlear regions to the FFR. In pilot studies this noise level was found to nearly completely mask the FFR to the AM tones when the noise was bandpass filtered between 0.4 and 8 kHz. All noise bands were independent between the two ears.

One thousand sweeps for each combination of stimulus configuration and MD were collected. Half sweeps were collected with the stimulus presented in rarefaction polarity, and half were collected with the stimulus presented in condensation polarity. FFRs were collected in four blocks of 2000 trials. On each block, 250 trials per stimulus configuration and MD were presented in a random order. The inter-stimulus interval was jittered between 25 and 75 ms.

### 2.6 FFR Processing

The triggers marking the onset of the stimuli in the EEG recordings were delayed by 0.9 ms to compensate for the acoustic delay introduced by the earphone tubes. The continuous EEG recordings were bandpass filtered offline between 0.06 and 1 kHz using a 256-taps FIR filter. The data from the HF electrode were referenced to the C7, linked earlobes (LERL), linked mastoids (LMST), and linked tiptrodes (LTPR) electrodes to obtain four different montages. The recordings were then segmented from -5 to 450 ms relative to stimulus onset, and baseline corrected using a 5-ms baseline. The segments for each stimulus were averaged using an iterative-weighted average (Riedel et al., 2001). Addition, and subtraction waveforms were obtained by respectively summing, or subtracting the averages for the stimuli presented in opposite polarities (rarefaction, condensation). The resulting waveforms were windowed using a hamming window, and the waveform spectra were computed via fast Fourier transforms (FFTs). For each of the target signal frequencies the level of the signal and of the noise were estimated from the FFT obtained from the corresponding segmentation procedure. The signal level was estimated by the power at the FFT bin closest to the signal frequency. The noise level was estimated by summing the power of six bins above and six bins below the signal bin, but excluding the two bins immediately below, and the two bins immediately above the signal bin to minimize the effects of spectral leakage on the noise estimate. The signal and noise levels were used to compute SNRs for the envelope (ENV) frequencies of both carriers from the addition waveforms, and for the temporal fine structure (TFS) frequencies (CF−MF, CF, and CF+MF) of the low-frequency carrier from the subtraction waveforms (Goblick, Pfeiffer, 1969, Greenberg et al., 1987). The TFS frequencies of the high-frequency carrier were too high to elicit FFRs (Krishnan, 2007).

The average SNR of the CF+MF component was close to zero for all electrode montages, probably because this component was not sufficiently resolved at the level of the basilar membrane to generate a FFR, and the data of this component were discarded from further analyses. Averaging across stimuli and the remaining ENV and TFS components, the SNR differences between the four montages were  < 1 dB. Given that SNR differences between montages were small, and because we are not aware of data suggesting that one montage would be optimal for detecting CS over the other montages, the data from all montages were used and modeled jointly in the statistical analyses. For brevity, only the average across-montage data, and the main effects estimated across montages, will be presented and discussed in the paper. Montage-specific data and effects are presented in the SM.

### 2.7 FFR Group delay estimation

FFR latencies were estimated via group delay using the algorithm described by King et al. (2016). The different MFs of the AM tones used to elicit the FFRs generated four components with closely spaced frequencies that could theoretically be used for group delay estimation in three frequency regions: the frequency region of the MF ( ~ 100 Hz) which could be used to estimate the ENV latency, and the frequency regions of the lower (CF−MF), and upper (CF+MF) side bands of the LCF AM tone, which could be used to estimate the TFS latency.

Only components with an SNR  >  6.64 dB were used for group delay estimation. These components have  < 1% probability to occur under the null hypothesis of equivalent signal and noise power according to an *F*_2,2*m*_ test (Dobie and Wilson, 1996), where *m* is the number of bins used to compute the noise power (m=12 in this case). Group delay estimates were calculated only if all four components for each target frequency passed the SNR criterion. Group delay estimates for the ENV and TFS components were respectively derived from the addition, and subtraction waveforms.

The King et al. (2016) algorithm estimates group delay by selecting the best least-square fit of components phases vs frequencies among the fits obtained by all possible unwrappings of the phases that are consistent with group delays in a given latency range. For this study, all phase unwrappings leading to latencies in the 0–30 ms range were considered. Only fits with a mean squared error (MSE)  < 0.01 were retained. All other details of the unwrapping and fitting procedure that are not explicitly mentioned here were the same as those described in King et al. (2016), and we refer readers to that paper for a full description of the algorithm. Given the above constraints, missing latency data could be due to either an insufficient number of components passing the minimum SNR criterion, or to best fits failing the maximum MSE criterion. Because more than 75% of the latency data were missing for the CF−MF TFS component (and the CF+MF component had already been excluded from further analyses due to its low SNR), the TFS latency data were not analyzed further. For the ENV component  ~  28% of latency data points were missing.

### 2.8 Noise exposure

Lifetime noise exposure was estimated via the structured interview developed by Lutman et al. (2008), which estimates the duration and level of noise exposure for a range of activities. One unit of noise exposure calculated via the interview corresponds to an eight hour daily exposure, for five days a week, for a year, to a noise level of 90 dBA. The estimated noise exposure was summed across all activities (occupational or recreational) to estimate the total cumulative noise exposure (TCNE). For the analyses the TCNE was log-transformed using base 10, so that a unit difference in the log_10_-transformed TCNE corresponds to a tenfold difference in noise exposure energy. Further details of the noise exposure interview are presented in the SM.

### 2.9 Audiometric thresholds

Audiometric thresholds were measured for pure tones at octave frequencies from 0.125 to 8 kHz (clinical frequency range) as well as for pure tones at 12 and 16 kHz (extended high-frequency range) using a two-interval two-alternative forced-choice task with an adaptive two-down one-up transformed up-down procedure tracking the 70.7% correct point on the psychometric function (Levitt, 1971). Details of the procedure are presented in the SM.

### 2.10 Statistical analyses

All analyses were performed using Bayesian models implemented by Markov Chain Monte Carlo (MCMC) simulations using JAGS (Plummer, 2003) and R (R Core Team, 2020). The data were analyzed using robust mixed-effect multiple regression models which included both categorical and continuous predictors, as well as random effects of subjects. Details of the models are given in the SM, and the model code is available at https://doi.org/10.17605/OSF.IO/S3BD9.

Effects were summarized by 99% credibility intervals (CIs) of the posterior distribution of the parameter of interest. These indicate that, according to the model, the parameter has a 99% probability of being enclosed within the bounds of the interval. The use of CIs to summarize the results of the study is in line with calls from different schools of statistical thought for a shift from crude null hypothesis testing to explicit estimation of the size of parameters of interest, and the uncertainty of these estimates (Gardner, Altman, 1986, Kruschke and Liddell, 2018, McShane, Gal, Gelman, Robert, Tackett, 2019, Schmidt, 1996). This approach emphasizes the idea that statistical results provide graded evidence, or different degrees of (un)certainty regarding a hypothesis, avoids conflating statistical significance with practical and/or theoretical significance, and acknowledges that single studies can rarely provide on their own conclusive evidence for or against an effect. Nonetheless, it is difficult to summarize succinctly the results of a large-scale study without making some categorical statements. For this reason we will refer to parameters whose 99% CIs excludes zero as being credibly different from zero to highlight the most salient findings, but we will also emphasize the size and uncertainty of effect estimates.

#### 2.10.1 ABR Wave amplitudes model

Log wave amplitude was used as the dependent variable (Bramhall, Konrad-Martin, McMillan, Griest, 2017, Bramhall, McMillan, Gallun, Konrad-Martin, 2019, Carcagno, Di Battista, Plack, 2019). Wave I and wave V amplitudes were modeled jointly. The data from the HP masking noise conditions were modeled separately from the data collected in quiet. The independent variables for the HP masking noise model included wave (I or V), click level (80 or 105 dB ppeSPL), montage (HF-ITPR or HF-IERL), sex, age, average pure tone thresholds between 0.5 and 2 kHz (PTA${}_{0.5-2}$), and log_10_TCNE, as well as a series of interaction terms between these predictors. All model terms and priors are listed in Table S3 of the SM. PTA${}_{0.5-2}$ was included as a predictor because even though all participants had near-normal audiometric thresholds below 4 kHz, there were residual threshold differences across the age range, with higher thresholds in this low-frequency region with increasing age. Sex was included as a predictor because of its known association with ABR wave amplitudes (Don et al., 1993). The model for ABR amplitudes in quiet was similar to the one for ABR amplitudes in HP masking noise, but included average pure tone thresholds between 4 and 12 kHz (PTA${}_{4-12}$) as an additional predictor (and the interactions of PTA${}_{4-12}$ with other predictors) to account for high-frequency audiometric losses. Model terms for ABR amplitudes in quiet are listed in Table S4.

A censored data analysis (Kruschke, 2014, Lunn, Jackson, Best, Thomas, Spiegelhalter, 2012) was used to deal with missing amplitude data due to undetectable peaks: all peak amplitudes that were missing were coded as being  < 0.38 nV, which was the lowest recorded amplitude value in the dataset. The Bayesian model then jointly estimated the value of the missing data and the likelihood function of the model given the constraints that the missing data are positive (constraint enforced by the log model) and  < 0.38 nV. Similar approaches have been used before in the analysis of ABR (Bramhall et al., 2019b) or electrocochleography data (Gajewski et al., 2004).

#### 2.10.2 ABR Wave latencies model

Wave I and wave V peak latencies, in ms, were modeled jointly. The statistical model for ABR wave latencies included the same predictors used in the model for ABR wave amplitudes. Unlike the wave amplitudes model, no censoring was used for ABR wave latencies, because no assumptions could be made on the distribution of the actual latency values of the missing latency datapoints. Model terms for ABR latencies in HP noise, and in quiet are listed in Tables S5, and S6, respectively.

#### 2.10.3 Amplitude and latency FFR ENV models

For FFR amplitudes the dependent variables consisted of the FFR SNR at the ENV frequencies of both carriers; these were modeled jointly. The independent variables included CF (0.6, or 2 kHz), MD (70%, or 100%), montage, age, average pure tone thresholds between 1 and 2 kHz (PTA${}_{1-2}$), log_10_TCNE, and years of musical experience, as well as a series of interaction terms between these predictors, which are listed in Table S7. Because four different MFs were used to allow the estimation of group delay, for each combination of CF X MD four measurements were available per participant. These four measurements were averaged before being entered into the analysis. The choice of frequencies included in the PTA${}_{1-2}$ predictor was dictated by the fact that the highest contributions to FFRs come from cochlear places higher than an octave above the center frequency (Ananthanarayan, Durrant, 1992, Dau, 2003), and contributions above 3 kHz were masked by the HP noise. Musical experience was added as a predictor to the FFR models because it has been found to be associated with FFR metrics (e.g. Bidelman, Gandour, Krishnan, 2011, Wong, Skoe, Russo, Dees, Kraus, 2007). Because the distribution of the number of years of musical experience was right skewed, a cube root transformation was applied to this variable before statistical analyses; this transformed variable will hereafter be referred to as MUS.

The model structure of the FFR latencies model was the same as that of the FFR amplitudes model just described. Model terms are listed in Table S9. The dependent variables for the latencies model consisted of the latencies estimated by the group delay algorithm.

#### 2.10.4 FFR TFS Model

For each participant 16 TFS measurements were available, given by 4 MFs X 2 MDs X 2 frequencies (CF−MF, and CF). None of these factors was of interest in the analysis, so the 16 measurements were averaged before being entered into the model. The FFR TFS model was essentially the same as the FFR ENV model, except for the fact that it did not include as independent variables CF (because only the LCF TFS responses were used), MD, and the interactions of these variables with the other independent variables. The model terms are shown in Table S8.

## 3 Results

### 3.1 Predictor variables

**Fig. 2:**
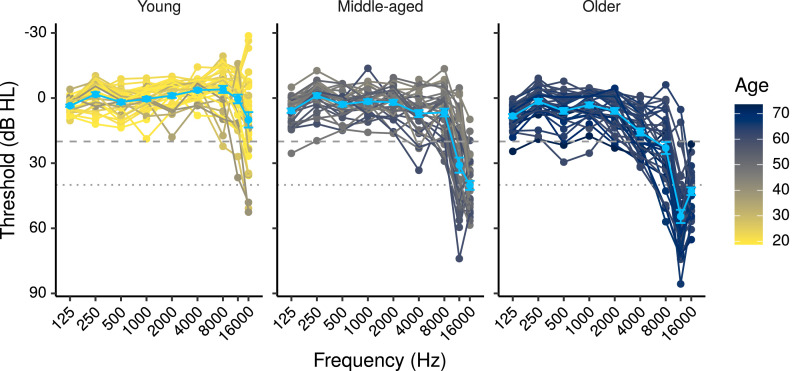
Audiometric thresholds for the study participants. The light blue points plot mean thresholds  ±  1 standard error of the mean (s.e.m.) for each age group. The dashed and dotted lines mark respectively 20, and 40 dB HL.

**Fig. 3:**
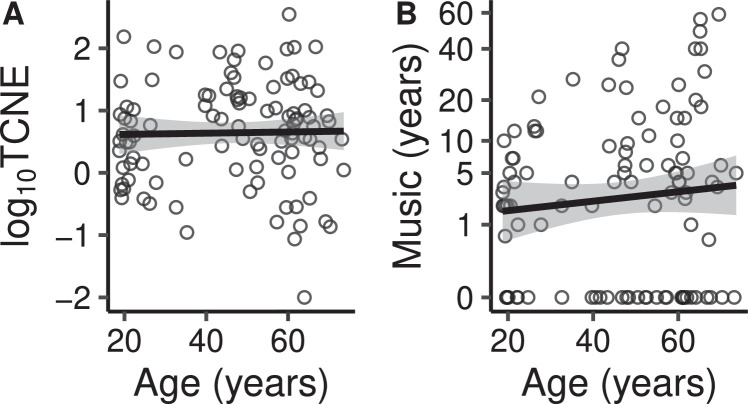
**A.** Total cumulative lifetime noise exposure as a function of age. A unit difference in the base 10 logarithmic y axis of the figure corresponds to a tenfold difference in noise exposure energy. **B.** Years of musical experience as a function of age. The y axis is shown on a cube root scale (years are displayed in their original unit).

Fig. 2 shows the audiometric thresholds for the participants included in the study, while Fig. 3A, and 3B show, respectively, log_10_TCNE, and the number of years of musical experience as a function of age. Table 1 shows correlations among predictor variables used in this study, along with 99% CIs computed with a Bayesian model based on that of Lee and Wagenmakers (2014, chap. 5, see SM for details). Not all of the predictors shown in the table were used in all models (e.g. PTA_4–12_ was only included for the ABR model in quiet in which high-frequency cochlear contributions were not noise masked), for details of the predictors used in each model see Section 2.10. For simplicity the correlations of the PTA_1–2_ variable used in the FFR models have been omitted from the table because they were very similar to the correlations of the PTA_0.5–2_ variable.

**Table 1:** Matrix of correlation coefficients among the predictor variables. 99% CIs are shown in brackets.

	PTA_0.5–2_	PTA_4–12_	Log_10_TCNE	Music $\sqrt[{3}]{Y}$
Age	0.37 (0.13–0.57)	0.84 (0.74–0.90)	0.02 (-0.23–0.28)	0.1 (-0.16–0.34)
PTA_0.5–2_		0.49 (0.27–0.67)	-0.11 (-0.35–0.15)	-0.03 (-0.28–0.23)
PTA_4–12_			0.06 (-0.21–0.29)	-0.06 (-0.31–0.19)
Log_10_TCNE				-0.01 (-0.26–0.25)

As expected, age was highly correlated with PTA${}_{4-12}$.^1^ Despite the fact that all listeners had normal or near normal hearing up to 2 kHz, age had a moderate correlation with PTA${}_{0.5-2}$. PTA${}_{0.5-2}$ and PTA${}_{4-12}$ were moderately correlated. None of the other predictors had sizeable correlations. It is notable that in this sample, age was not associated with increased log_10_TCNE. This may reflect geographical or historical peculiarities of the participants sample, as well as the fact that they were a self-selected sample of volunteers from the general population. For most listeners the major contributor to TCNE was recreational noise exposure, and this tended to be concentrated in their youth years. Nonetheless TCNE had a large spread across the sample, varying over more than three orders of magnitude.

### 3.2 ABR Wave amplitudes

**Fig. 4:**
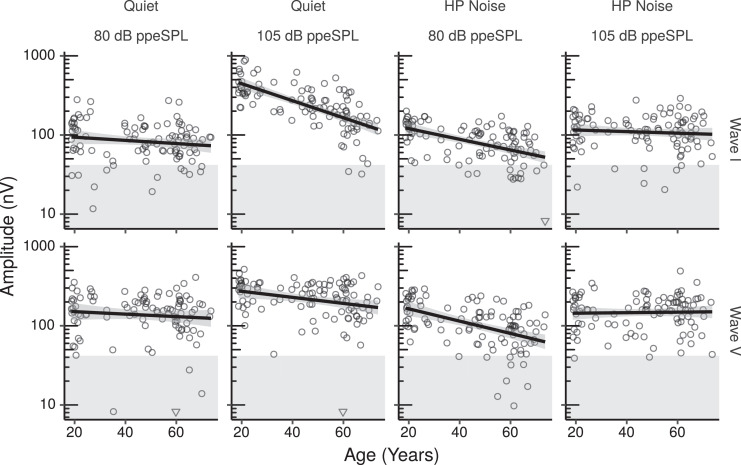
ABR wave I and V amplitudes by age for the HF-ITPR montage. The inverted triangles represent data points for which the peak-trough amplitude could not be measured. These data points were modeled as having an amplitude lower than the lowest recorded peak-trough amplitude in the dataset through a censored analysis. Each panel shows a least squares line fit of wave amplitude by age with 95% confidence intervals as a visual aid. The slope for the effect of age estimated by the Bayesian multiple regression model is not the same as that shown in the figure.

ABR grand averages for the HF-ITPR montage are shown in Fig. S1 separately for each age group. It should be emphasized that although the grand averages are shown in this figure as a function of discrete age groups for illustrative purposes, age was used as a continuous variable in all the analyses. Fig. 4 shows the ABR wave I and V amplitudes measured for each participant in each condition as a function of age with the HF-ITPR montage. For some conditions wave amplitudes for some participants were rather low. In order to estimate the noise floor we ran the same peak-picking algorithm used to find wave I on an equivalent time window in the pre-stimulus baseline. The geometric mean of the peak-trough amplitude of this dummy wave in the pre-stimulus baseline was 41.87 nV (geometric sd=1.91). Note that this is likely a slight overestimate of the mean “noise floor”, because the average excluded 3.9% of the datapoints for which the peak-trough amplitude of the dummy wave could not be estimated. The mean noise floor is denoted in the figure by the shaded gray area. For all conditions, most of the datapoints fell well above this average noise floor.

**Fig. 5:**
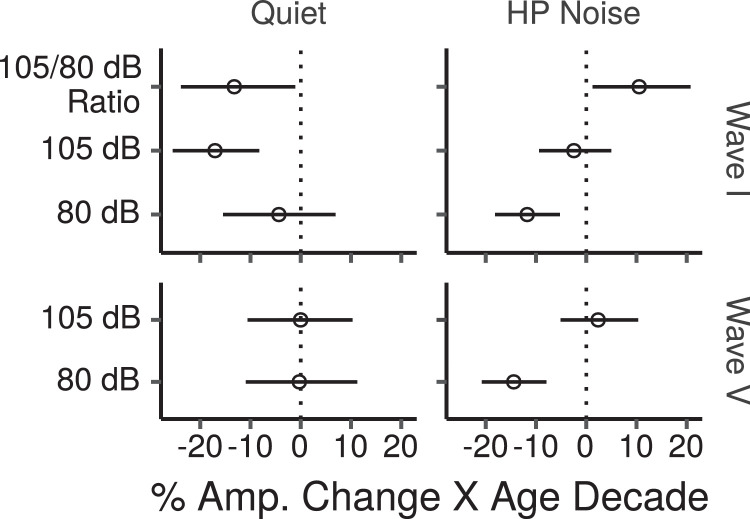
Posterior medians (circles) and 99% credibility intervals for the effects of age on ABR wave I and V amplitudes estimated by the Bayesian multiple regression models for the HF-ITPR montage. Effects are plotted as percentage amplitude change for an age increase of 10 years.

From Fig. 4 it is apparent that, in quiet, wave I amplitude at the high stimulus level decreased considerably as age increased, while in HP noise, there was no apparent age-related decrease in wave I amplitude. The opposite pattern occurred at the low stimulus level, with an apparent age-related decrease of wave I amplitude in HP noise, and little change with age in quiet. Fig. 5 shows the effects of age on wave amplitudes estimated by the multiple regression models for the ABR in quiet and in HP masking noise. The results for the ABR in quiet indicate a credible decrease in wave I amplitude as a function of age at the high stimulus level after controlling for the effect of covariates. The median of the posterior distribution for this decrease was  ~ 17% per age decade (CI: 8 – 26%). Importantly there was also a credible age-related decrease of the wave I amplitude ratio between the high and the low stimulus levels, with a posterior median of  ~ 13%, and CIs ranging from about 1 to 24%. There was no evidence of age-related changes in wave V at either stimulus level.

The results for the HP noise condition show a credible age-related decrease of wave V at the low stimulus level, with a posterior median of  ~ 14% per age decade (CI: 8 – 21%). For wave I at the low stimulus level there was also a credible age-related decrease of similar magnitude. The results do not provide evidence of an age-related decrease of wave I or wave V amplitudes at the high stimulus level. The wave I amplitude ratio between the high and the low stimulus levels, rather than decreasing, show a credible age-related increase. It should be pointed out that this effect is unlikely to be due to age-related OHCs dysfunction, given that the effects of low-frequency audiometric thresholds were partialed out. Additionally, this effect cannot be attributed to age-related medial olivocochlear reflex deficits, given that the reflex onset is of  ~ 25 ms (Backus, Guinan, 2006, Lopez-Poveda, 2018), and the duration of the stimulus, including the HP masking noise, was 20 ms. There is also no evidence that the wave I/V amplitude ratio, which is difficult to interpret for broadband ABRs, but has been suggested as a useful normalized CS metric for frequency-restricted ABRs (Bharadwaj et al., 2019), was affected by age at high stimulus levels in HP noise (CI: -13 – 4% change per age decade).

It is notable that while wave amplitudes in quiet increased considerably as a function of stimulus level, in HP noise there was no apparent increase of wave amplitudes with increasing stimulus level for the young participants. The estimated % growth from 80 to 105 dB ppeSPL at age 20 (with PTA${}_{0.5-2}$ and log_10_TCNE set at their mean across the age range) was 1 (CI: -25 – 33) for wave I, and -8 (CI: -32 – 23) for wave V. While at age 70 it was 67 (CI: 23 – 117) for wave I, and 125 (CI: 66 – 199) for wave V. Possible implications of this finding will be discussed later.

The effects of PTA${}_{0.5-2},$ and PTA${}_{4-12}$ on ABR wave amplitudes estimated by the model are shown in Figs. S2 and S3, while the effects of log_10_TCNE are shown in Fig. S4. None of these effects was credibly different from zero, although the CIs suggest caution in interpreting these as null effects. The effects of sex are shown in Fig. S5. Waves I and V in quiet at the low stimulus level had a credibly larger amplitude for females compared to males. Trends in the same direction were also present for wave V in HP noise at the low stimulus level, and for wave I at the high stimulus level both in quiet and in HP noise.

### 3.3 ABR Wave latencies

**Fig. 6:**
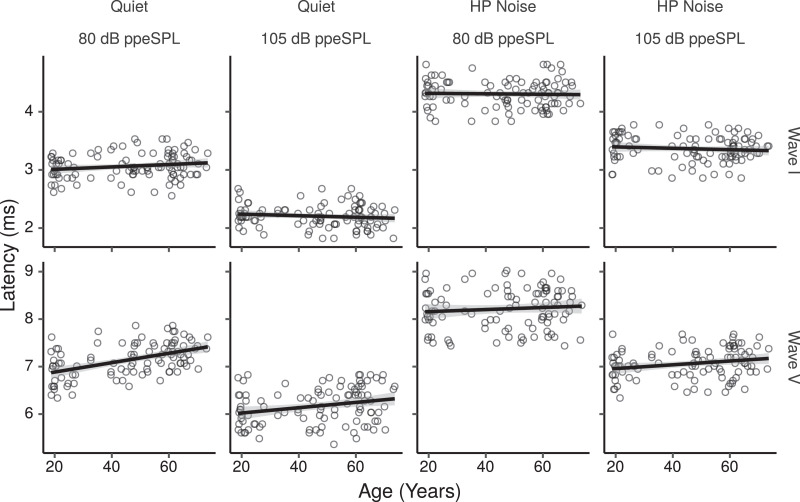
ABR wave I and V latencies by age for the HF-ITPR montage. Each panel shows a least squares line fit of wave latency by age with 95% confidence intervals as a visual aid. The slope for the effect of age estimated by the Bayesian multiple regression model is not the same as that shown in the figure.

**Fig. 7:**
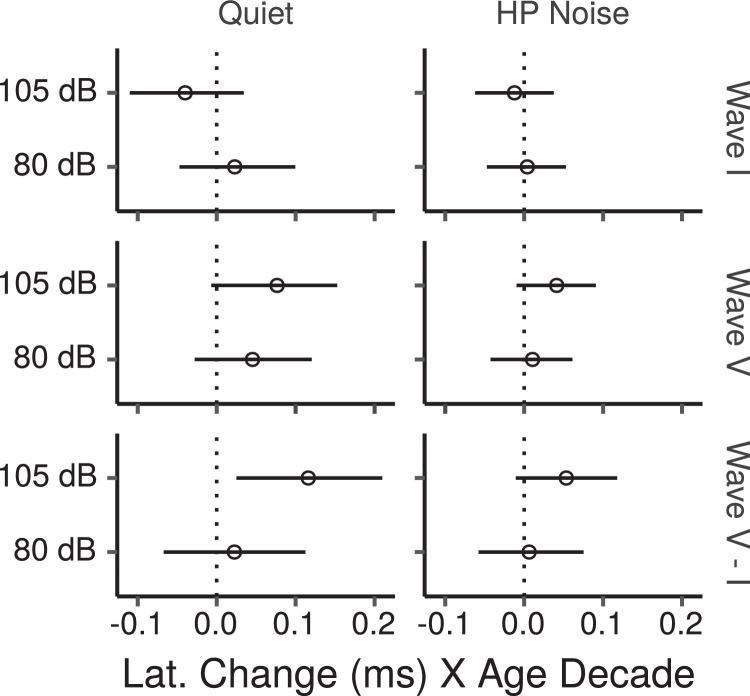
Posterior medians (circles) and 99% credibility intervals for the effects of age on ABR wave I and V latencies estimated by the Bayesian multiple regression model for the HF-ITPR montage. The bottom row shows the age effect for the wave I–V interpeak latencies. Effects are plotted as latency change for an age increase of 10 years.

Fig. 6 shows the ABR wave latencies measured for each participant in each condition as a function of age for the HF-ITPR montage. The CIs for the effect of age on wave latencies are shown in Fig. 7. In quiet there was a trend for wave V latencies to increase at both low, and high stimulus levels. There were no notable trends for age effects on wave I latencies, except for a tendency to shorter latencies at the high level. There was a credible age-related increase in wave I–V interpeak latency for the high stimulus level.

The model estimates for wave V latencies in HP noise show a trend for age-related latency increases at the high stimulus level, and for age-related increases of the wave I–V interpeak latency. There was no evidence of age effects at the low stimulus level for either of the waves or for the I–V interpeak latency.

The effects of PTA${}_{0.5-2},$ and PTA${}_{4-12}$ on ABR wave latencies estimated by the model are shown in Figs. S6 and S7. In quiet there was a weak trend for latency increases with increases in PTA${}_{0.5-2}$ for wave I at the low stimulus level. This was reflected in a trend for shorter wave I–V interpeak latencies. There was also a trend for latency increases with increasing PTA${}_{4-12}$ for wave V at the low stimulus level, which was reflected in a trend for longer wave I–V interpeak latencies. No notable trends were present in HP noise, except for weak trends towards increasing wave V latencies, and decreasing wave I latencies at the low stimulus level, which were reflected in a trend for longer wave I–V interpeak latencies.

The effects of log_10_TCNE are shown in Fig. S8. None of these effects was credibly different from zero, and no notable trends were observed. The effects of sex are shown in Fig. S9. For wave I no notable trends were observed. For wave V in quiet females had credibly shorter latencies than males at both stimulus levels. A trend for shorter wave V latencies for females in the HP noise condition was also present at the high stimulus level.

### 3.4 FFR ENV SNR

**Fig. 8:**
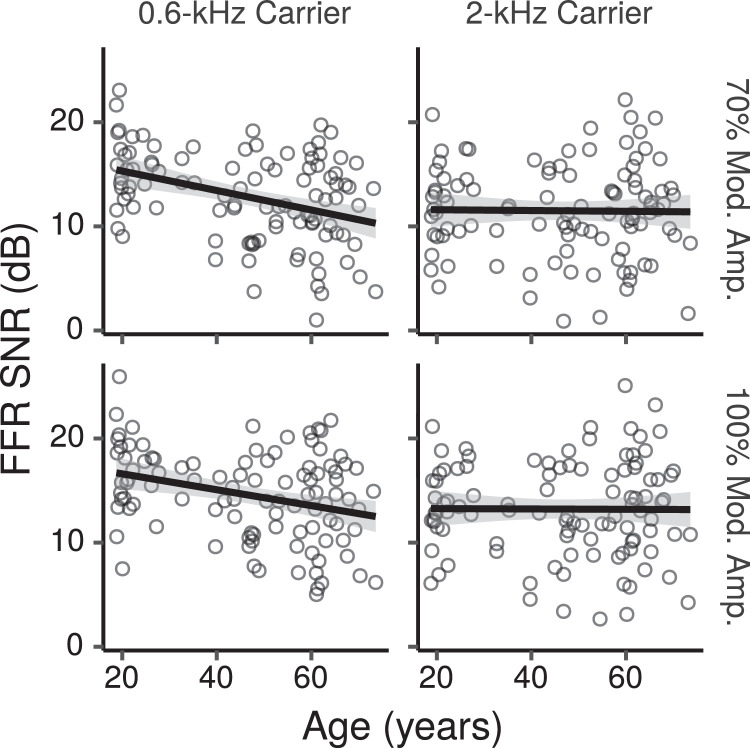
Across-montage average FFR ENV SNR by age. Each panel shows a least squares line fit of FFR SNR by age with 95% confidence intervals as a visual aid. The slope for the effect of age estimated by the Bayesian multiple regression model is not the same as that shown in the figure.

**Fig. 9:**
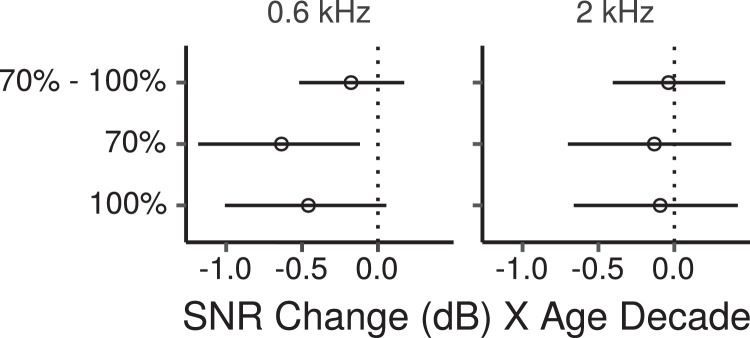
Posterior medians (circles) and 99% credibility intervals for the main effects (across montages) of age on FFR ENV SNR estimated by the Bayesian multiple regression model. The top row shows the effect difference between the 70% and 100% MD. Effects are plotted as an SNR change for an age increase of 10 years.

Fig. 8 shows the across-montage average FFR ENV SNR measured for each participant in each condition as a function of age. From the figure SNRs appear to decrease with increasing age for the 0.6-kHz CF, while they appear to change little for the 2-kHz CF. Fig. 9 shows the CIs for the main effects of age on FFR ENV SNR across montages. For the 0.6-kHz CF there was a credible decrease in ENV SNR with age for the 70% MD with a posterior median of -0.6 dB per age decade (CI: -1.2 – -0.1), and a trend in the same direction for the 100% MD (posterior median -0.5 dB, CI: -1 – 0.1). The difference for the effect of age between the 70% and 100% MDs had a posterior median of -0.2 dB per age decade (CI: -0.5 – 0.2). Thus there was not much evidence of a greater age effect at the lower MD, as predicted by the CS hypothesis, although the trend was in that direction. There was no credible age-related decrease for the 2-kHz CF at either MD, with the lower and upper bounds of the CIs ranging from  ~  -0.7 to 0.4 dB per age decade.

The effects of PTA${}_{1-2}$ on ENV SNR are shown in Fig. S10. There was a credible decrease in ENV SNR with increasing PTA${}_{1-2}$ for the 0.6-kHz stimulus at both MDs, with posterior medians of  ~  0.2 dB per dB of hearing loss (CIs  ~  -0.3 – -0.03). For the 2-kHz stimulus instead there was a trend for the SNR to increase with increasing PTA${}_{1-2}$ (posterior medians  ~  0.1 dB, CIs:  ~  -0.06 – 0.24).

The effects of log_10_TCNE are shown in Fig. S11. There were no credible changes in ENV SNR as a function of log_10_TCNE, with posterior medians for the effects at either CF and MD close to zero, and CIs compatible with changes of at most  ~  ± 1 dB for differences in lifetime noise exposure of a factor of 10.

The effect of MUS, which was estimated only across CFs and MDs, had a posterior median of 0.3 dB (CI: -0.3 – 1) per cubic root of years of musical experience.

### 3.5 FFR TFS SNR

**Fig. 10:**
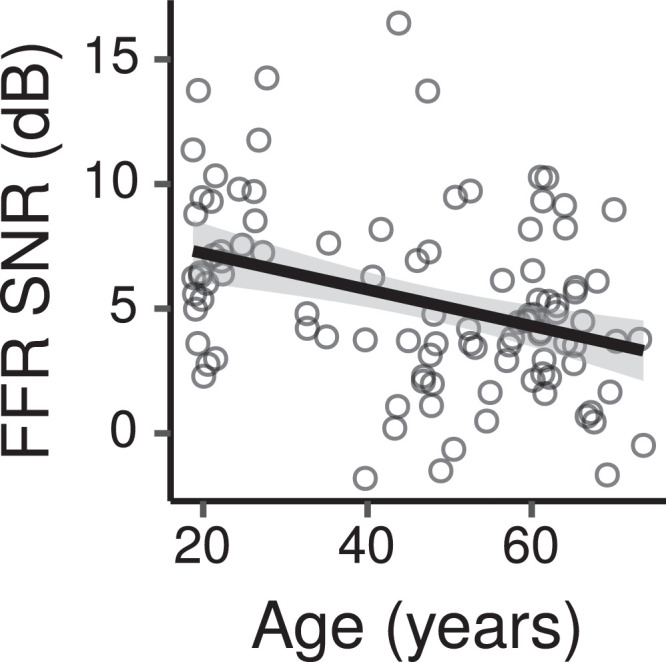
Across-montage average FFR TFS SNR by age. The figure shows a least squares line fit of FFR SNR by age with 95% confidence intervals as a visual aid. The slope for the effect of age estimated by the Bayesian multiple regression model is not the same as that shown in the figure.

Fig. 10 shows the across-montage average FFR TFS SNR measured for each participant in each condition as a function of age. From this figure the TFS SNR appears to decrease with increasing age. The CIs for the effect of age indicate a credible decrease in SNR with age, with a posterior median of -0.6 dB per age decade (CI: -1.1 – -0.04).

There were no credible effects of PTA${}_{1-2}$ (CI: -0.1 – 0.1), log_10_TCNE (CI: -1.6 – 0.3), or MUS (CI: -0.3 – 1.02) on TFS SNR.

### 3.6 FFR ENV Latency

**Fig. 11:**
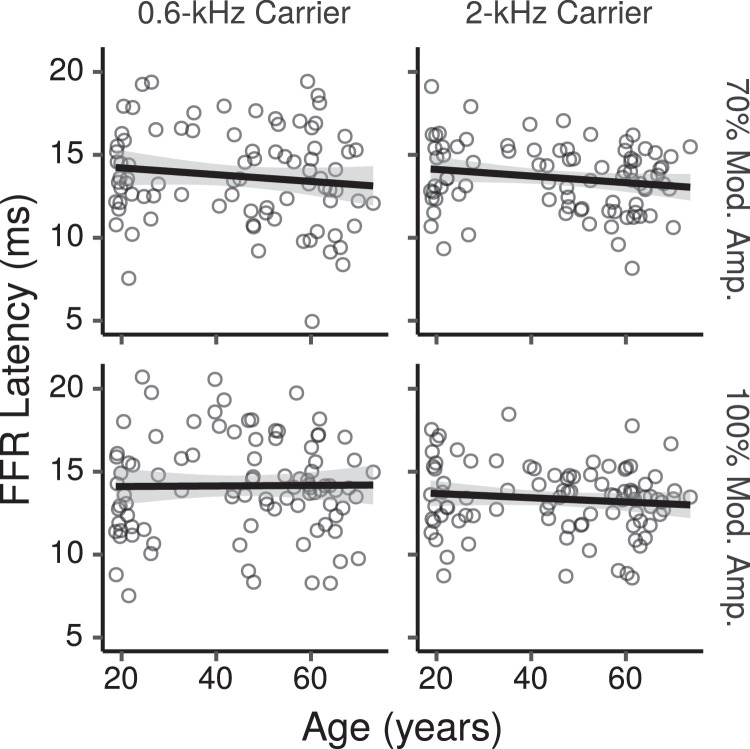
Across-montage average FFR ENV latency by age. Each panel shows a least squares line fit of FFR latency by age with 95% confidence intervals as a visual aid. The slope for the effect of age estimated by the Bayesian multiple regression model is not the same as that shown in the figure.

Fig. 11 shows the across-montage average FFR ENV latency estimates computed for each participant in each condition as a function of age. Average latency estimates were between 13 and 14 ms in different stimulus conditions. Latencies did not appear to change greatly with age.

**Fig. 12:**
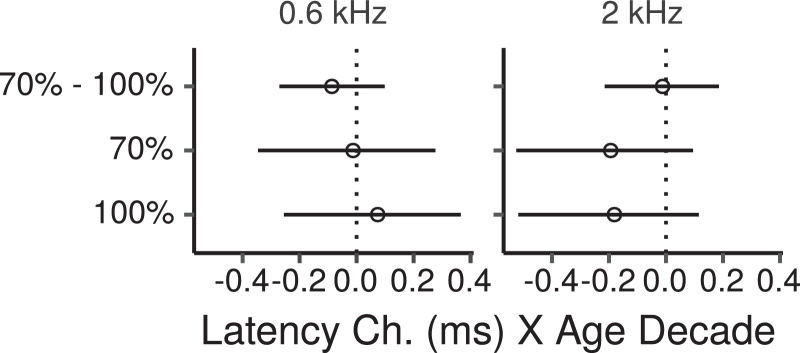
Posterior medians (circles) and 99% credibility intervals for the main effects (across montages) of age on FFR ENV latency estimated by the Bayesian multiple regression model. The top row shows the effect difference between the 70% and 100% MD. Effects are plotted as a latency change, in ms, for an age increase of 10 years.

The CIs for the main effect of age on FFR ENV latencies across montages are shown in Fig. 12. The CIs do not provide evidence of age effects for the 0.6-kHz CF, with posterior medians changes close to zero, and CIs within  ~  ± 0.4 ms. For the 2-kHz CF there were trends for shorter latencies with increasing age, with posterior medians of  ~  -0.19 ms per age decade, and CIs with lower and upper limits of  ~  -0.52, and 0.1 ms, respectively.

The effects of PTA${}_{1-2}$ (Fig. S12), log_10_TCNE (Fig. S13), and MUS (CI: -0.35 – 0.48) were not credibly different from zero and did not show notable trends.

## 4 Discussion

### 4.1 ABR Measures

ABR wave I responses in quiet showed an age-related decrease at the high stimulus levels; this decrease was greater at high than at low stimulus levels, as shown by the age-related reduction of the wave I 105/80 dB ppeSPL ratio, and was present despite partialing out potential effects of age-related audiometric threshold shifts in low and high frequency regions. This result is in line with those obtained by Johannesen et al. (2019), and Grose et al. (2019), and is consistent with the predicted effects of CS. However, as we have argued in the Introduction, this result does not provide compelling evidence for CS due to differences in cochlear frequency regions generating wave I at low and high stimulus levels, and potential difficulties in distinguishing effects due to CS from potential effects of age-related high-frequency hearing loss. A shallower growth of wave I amplitude with age, obtained with HP masking noise so that responses are restricted to a low frequency region where thresholds are near normal across the age range, would provide more convincing evidence of age-related CS. However, the results in HP noise did not follow this prediction: we found no evidence of age-related wave I reductions at high stimulus levels, while such age-related reductions occurred at low stimulus levels, unexpectedly leading to a steeper, rather than the shallower wave I amplitude growth with age that is predicted by the CS hypothesis.

Wave I amplitudes in the HP noise condition did not grow with level for young listeners. This response saturation for wave I was observed by Eggermont and Don (1980) at low-frequency derived bands, but its origins are unclear. This saturation effect may have limited our ability to observe age-related decreases at high stimulus levels. Some neurophysiological evidence suggests that only H-SR and M-SR fibers contribute to ABR wave I, while L-SR fibers do not because of the variability of their first-spike latency (Bourien et al., 2014). Thus, a possible explanation for the fact that wave I responses did not grow with level for young listeners is that the 80 dB ppeSPL stimulus was sufficiently intense to saturate H-SR and M-SR fibers generating the response. However, the hypothesis that the same population of saturated fibers was responding to the low and high level stimuli does not fit with the observed greater age-related wave I reduction at the low stimulus level. Given that age-related wave I reductions were greater at the low stimulus level, either the population of fibers generating the response was different (e.g. fibers with different spontaneous rates, or fibers responding to different cochlear places), or the response of the same population of fibers was modulated by level.

The reason for the greater age-related reduction of wave I at low than at high stimulus levels in HP noise is unclear. A selective age-related deficit of H-SR fibers seems contrary to the neurophysiological evidence of greater susceptibility of L/M-SR fibers to CS documented by neurophysiological studies in non-human animals, and thus unlikely, although it cannot be excluded. Although a selective age-related deficit of H-SR fibers would predict greater age-related wave I reductions at low than at high stimulus levels, it would predict some age-related wave I reductions at high stimulus levels. Our results do not provide supporting evidence for such reductions at high stimulus levels, but they are not inconsistent with them because the CIs are compatible with the presence of age-related reductions at high stimulus levels of up to  ~ 10% per age decade.

An alternative explanation for the greater age-related wave I reduction at low than high stimulus levels is that the responses at the two levels were partly originating from different cochlear places within the low-frequency band where the response was restricted. Eggermont and Don (1980) found that the relative contribution that different 1-octave frequency bands make to the unmasked wave I response is level dependent. At high levels the contributions of high-frequency bands become larger than those of low-frequency bands. This effect is more obvious when comparing a 0.5-kHz to a 4-kHz (or higher) band, but smaller level-dependent effects are already apparent when comparing an 0.5-kHz to a 2-kHz band. Hence, given that the click used in the current study spanned a relatively wide region (bandpass cutoffs: 0.35–3 kHz), it is possible that the age effect found at the low level reflects greater sensorineural deficits at more apical cochlear sites. However, it is not clear why sensorineural deficits would be greater at more apical sites. Future studies using the subtraction technique of responses high-pass masked at different cutoffs to isolate responses within more restricted regions (Don and Eggermont, 1978) could shed light on this issue.

While the results in HP noise seem at odds with the hypothesis that CS affected the low-frequency region stimulated by the highpass masked click, the results in quiet are consistent with, although they do not prove, the presence of CS in the frequency region from which the response in quiet originated. While ABRs in HP noise were restricted to a low-frequency cochlear region, the responses in quiet at high levels reflect the contribution of a wide cochlear region. Because of reduced traveling-wave dispersion at high-frequency cochlear regions, these responses are likely dominated by contributions of high-frequency cochlear channels. The different pattern of results obtained for the wave I 105/80 dB ppeSPL ratio in quiet and in HP noise could thus reflect differences in the degree of age-related CS at low and high frequency regions. Specifically our results are consistent with the presence of age-related CS in high-frequency cochlear regions, but not in low-frequency cochlear regions. Although the reason why CS would preferentially affect high-frequency cochlear regions is not clear, this interpretation of our results would be consistent with some (but not all) physiological studies. In particular, Schmiedt et al. (1996) found a drastic reduction in the proportion of L-SR fibers with characteristic frequencies  > 6 kHz in quiet-aged gerbils, but did not observe similar reductions for fibers with characteristic frequencies  < 6 kHz. Stamataki et al. (2006) found that the age-related loss of IHC synapses in C57BL/6J mice occurred mostly at basal sites, and Jiang et al. (2015) found that IHC ribbon losses in C57BL/6J mice with age started at basal sites before progressing to apical sites. Sergeyenko et al. (2013), however, found that IHC ribbon losses with increasing age in CBA/CaJ mice were initially greater at apical sites before progressing to basal sites. The differential involvement of apical vs basal sites may reflect differences between species, or between genetic strains within a species. In humans the age-related decline of SGN peripheral axons, although present throughout the cochlear length appears slightly larger towards the base (frequencies  > 1 kHz) than towards the apex (frequencies  < 1 kHz; Wu et al., 2019); a similar, although not significant trend of greater basal loss was observed for the age-related degeneration of SGN bodies (Viana et al., 2015).

Overall, no credible effects of age on wave I or wave V latencies were found. Given this, the increased wave I–V interpeak latency with age found at the high stimulus level in quiet, although suggestive of possible central neural conduction delays with age, should be interpreted cautiously. Previous large scale investigations have yielded mixed results, with some results supporting increased I–V interpeak latencies with age (Elberling, Parbo, 1987, Mitchell, Phillips, Trune, 1989), and some not (Konrad-Martin et al., 2012).

Although the focus of this study is on aging effects we will briefly comment on the effects of the other covariates. Estimates of the effects of lifetime noise exposure on wave amplitudes and latencies were generally close to zero and none was credibly different than zero. Although the results of this single study do not exclude the possibility of small or moderate effects of noise exposure on ABR measures (e.g. the CIs are compatible with wave amplitude reductions of  ~ 10% for a tenfold increase in lifetime noise exposure), they add to the results of the majority of studies on the topic, that have failed to find an effect of lifetime noise exposure on ABR amplitude and latency measures (see Bramhall, Beach, Epp, Le Prell, Lopez-Poveda, Plack, Schaette, Verhulst, Canlon, 2019, Le Prell, 2019, for reviews).

Audiometric threshold shifts in the current study may have been due to a mixture of OHC and IHC dysfunction (Johannesen et al., 2014). OHC dysfunction in the frequency region where the stimulus was presented would be expected to decrease wave I amplitudes especially at low levels, while OHC dysfunction in higher frequency regions should not greatly affect wave amplitudes. IHC dysfunction, on the other hand, could affect both on- and off-frequency responses. Although there were some trends for lower wave I amplitudes with increasing audiometric thresholds, no credible effects of audiometric thresholds on the ABR measures were found. The absence of sizeable effects of audiometric thresholds on ABR amplitude in this study may be due to the fact that audiometric thresholds shifts, when present, were at most mild (except in the extended high-frequency region). Furthermore, the strong correlation between high-frequency audiometric thresholds and age makes it difficult to isolate the effects of these two variables; this is reflected in the width of the CIs for the effects of high-frequency audiometric thresholds, that while not providing decisive evidence for such effects, are nonetheless compatible with their presence.

Similar considerations apply to audiometric effects on ABR latencies. For the stimuli in quiet, sloping high-frequency hearing losses, such as those of the older participants in the current study, may be expected to lead to delayed latencies at low stimulus levels because ABR contributions will tend to shift from the impaired basal sites towards the more apical sites with better preserved low-level sensitivity (Gorga et al., 1985). These effects, however, may have been too small to be reliably detected for a click with a level of 80 dB ppeSPL, and the mild high-frequency hearing losses of the older listeners in the current study (see simulations in Verhulst et al., 2016). For derived-band responses, the effect of OHC dysfunction changes both passive and active cochlear mechanics with opposite consequences on wave latencies at low stimulus levels: increased latency for the change in passive cochlear mechanics, and decreased latency for the wider auditory filters caused by the change in active cochlear mechanics (Don et al., 1998). The latter effect tends to dominate, leading to shorter ABR wave latencies, but only for hearing losses exceeding 20–30 dB (Don et al., 1998), which are larger than the hearing losses in the low-frequency region of the participants of the current study.

The trends for higher ABR wave amplitudes in quiet for females compared to males observed in the current study are consistent with previous reports (Don et al., 1993), and underline the importance of considering sex effects when designing and analyzing ABR experiments.

### 4.2 FFR Measures

The FFR SNR showed age-related decreases for both TFS and ENV components of the 0.6-kHz CF stimulus. These results are in agreement with several other studies showing age-related decreases in subcortical measures of neural phase locking to tones amplitude modulated at rates  ~ 100 Hz (Garrett, Verhulst, 2019, Grose, Mamo, Hall, 2009, Leigh-Paffenroth, Fowler, 2006). There was little evidence, however, that the age-related decreases for the ENV component were greater at shallow compared to deep MDs, as predicted by the CS hypothesis. A similar result was reported by Garrett and Verhulst (2019), who did not find differences in the slope of FFR SNR as a function of MD between a group of 22 young normal hearing listeners and a group of 23 older listeners with hearing loss. For an AM tone with a shallow MD, Encina-Llamas et al. (2019) found shallower FFR growth functions with level in four older hearing impaired listeners compared to nine young normal hearing listeners, while growth functions with level were similar for the two groups for an AM tone with a deeper MD. Although the results of this latter study seem consistent with a CS profile, considering its small sample size relative to the current study and that of Garrett and Verhulst (2019), the overall results of these studies do no provide much evidence that age-related FFR decreases are greater at low MDs. Moreover, the interpretation of the results of Garrett and Verhulst (2019) and Encina-Llamas et al. (2019) is complicated by the fact that neither study used HP noise to mask the contribution of high-frequency cochlear regions, nor estimated age effects while simultaneously controlling for audiometric threshold shifts. Simulations of auditory nerve activity run by Encina-Llamas et al. (2019) suggest that, without HP masking noise, off-frequency contributions tend to dominate the FFR, and detecting the effects of even a complete loss of L/M-SR fibers becomes difficult because of the large contribution of off-frequency H-SR fibers to the response.

Age effects were greater for the 0.6-kHz than for the 2-kHz CF (median posterior difference: 0.43 dB, CI: 0.17 – 0.7). Leigh-Paffenroth and Fowler (2006) similarly observed a greater reduction in the number of FFRs above the noise floor to a 0.5-kHz CF than to a 2-kHz CF in a group of 12 older participants compared to a group of 16 young participants. Grose et al. (2009), on the other hand, did not find that the reductions in FFR SNR in a group of 10 older listeners compared to a group of 10 young listeners, were significantly different between a 0.5-kHz and a 2-kHz AM tone. The reason for the across-CF differences found by Leigh-Paffenroth and Fowler (2006) and in the current study remains unclear^2^.

Our results do not provide evidence for an effect of lifetime noise exposure on FFR ENV and TFS SNRs. The results of the current study are not conclusive regarding the presence/absence of such effects, given that the CIs are compatible with effects of lifetime noise exposure (in either direction) of up to  ~  1.5 dB per tenfold difference in noise exposure. However, they add to the results of other large-scale studies (Prendergast et al., 2017, Prendergast, Couth, Millman, Guest, Kluk, Munro, Plack, 2019) that found that the effect of lifetime noise exposure on FFR SNR was close to zero.

The reduction of basilar membrane compression associated with OHC dysfunction is expected to lead to increased FFR responses to the envelope of AM tones. Although this effect was not credibly different from zero, FFR ENV SNR at the 2-kHz CF tended to increase with increasing PTA${}_{1-2},$ consistent with loss of basilar membrane compression caused by OHC dysfunction. At the 0.6-kHz CF, however, FFR ENV SNR credibly decreased with increasing PTA${}_{1-2}$. Although this decrease is opposite to what would be predicted by OHC dysfunction, it is compatible with IHC dysfunction.

It is likely that the effects of PTA${}_{1-2}$ observed at both CFs reflect a mixture of OHC and IHC dysfunction. Basilar membrane compression is likely reduced towards apical sites compared to basal sites (Robles and Ruggero, 2001), and this may explain why FFR ENV responses do not tend to increase with PTA${}_{1-2}$ at the lower CF. Alternatively, it is possible that the FFR ENV response to the lower CF was dominated by off-frequency contributions, which are not shaped by OHC function and have a linear, non-compressive response. Although we cannot exclude this hypothesis, simulation results from a recent study (Encina-Llamas et al., 2020) suggest that the presence of a tone with a higher CF, such as the 2-kHz tone in the current study, dramatically reduces the off-frequency responses to a tone at a lower CF, such as the 0.6-kHz CF tone. Therefore, a dominance of off-frequency contributions is unlikely to explain the absence of apparent OHC dysfunction effects on the 0.6-kHz CF results.

FFR ENV latency estimates via group delay in the current study were on average 4–5 ms longer than those estimated in a previous study (King et al., 2016). This may partly reflect the fact that, due to the use of HP masking noise, responses were restricted to apical cochlear regions with long traveling wave delays.

No credible effects of age were found on estimated FFR latencies. Although this result does not provide evidence of age-related increases of FFR ENV latencies, there are three reasons why it should be interpreted cautiously. One is the residual uncertainty of the estimates, with CIs compatible with changes of up to  ~  0.5 ms per age decade. The second is the fact that the missing data were more prevalent as age increased, and although the difference between age groups was not large (young: 22%, middle-aged: 29%, older: 34%), it may have biased the estimated effects of age on latencies if the reduced SNR that caused the data to be missing, is associated with longer or shorter latencies. The third factor to take into account is that the latency estimate assumes a single FFR source, but the recorded FFR may reflect multiple sources with different latencies interacting constructively or destructively (King et al., 2016). This third factor makes the interpretation of the data more difficult, and may explain the large spread of the latency estimates observed in the current study.

### 4.3 Conclusions

Overall, the ABR wave I and FFR ENV SNR results of this study obtained with HP masking noise do not provide evidence of age-related CS occurring in low-frequency regions ( ≲  3 kHz) with near-normal audiometric thresholds across the age range. However, the ABR wave I results in quiet are compatible with CS affecting higher frequency regions.

Although our results do not provide evidence of age-related CS in low-frequency regions, they do not warrant the stronger conclusion that age-related CS is not occurring in these frequency regions. As discussed in Section 4.1, the lack of ABR wave I growth with level in young listeners may have limited our ability to detect CS. For the FFR measure, there is uncertainty regarding the MF of AM tones at which effects of CS could be detected. CS effects in CBA/CaJ mice are largest for MF  ~  1 kHz (Shaheen et al., 2015), that reflect mostly auditory nerve activity, but some differences are apparent also at MFs  ~  100 Hz (Parthasarathy and Kujawa, 2018), that reflect mostly brainstem activity. It has been hypothesized that smaller CS effects are seen at lower AM rates because of compensatory mechanisms increasing gain at brainstem and cortical levels (Parthasarathy, Bartlett, Kujawa, 2019, Parthasarathy, Kujawa, 2018); however, there is evidence that these compensatory mechanisms may themselves decline with age (Möhrle et al., 2016). A recent study using transposed tones with a 4-kHz carrier failed to find age effects at higher modulation rates in the range of 240–285 Hz (Prendergast et al., 2019), suggesting that targeting higher MFs may not better reveal potential CS effects. FFRs in humans become more difficult to record at higher AM rates close to 1 kHz, but computational models of the human auditory periphery suggest that effects of CS on the FFR should be detectable at lower rates close to 100 Hz (Verhulst et al., 2018).

Although the specific CS profile of age-related reductions of WI_H_/WI_L_ and FFR_S_-FFR_D_ was not observed in the current study, several ABR and FFR measures showed age-related reductions that could not be accounted for by audiometric hearing losses. These may reflect sensorineural deficits other than CS (Caspary, Ling, Turner, Hughes, 2008, Ouda, Profant, Syka, 2015, Schmiedt, 2010). Alternatively, it is possible that age-related CS in humans does not follow the same profile as noise-induced CS in rodents, who show a predominant loss of L/M-SR fibers.

Although age-related synaptic loss in humans has been documented (Viana, O’Malley, Burgess, Jones, Oliveira, Santos, Merchant, Liberman, Liberman, 2015, Wu, Liberman, Bennett, de Gruttola, O’Malley, Liberman, 2019), it is not known whether this loss affects primarily L/M-SR fibers. As noted by Hickox et al. (2017), the association between the spontaneous rates of auditory-nerve fibers and their thresholds, which has been observed for a number of mammalian species, was not observed in a study on a non-human primate species (macaque; Joris et al., 2011). Therefore, it is not clear that the pathophysiological model of age-related CS affecting mainly fibers with high thresholds applies to humans.

Although the greater involvement of L/M-SR fibers has been shown to occur after noise-induced CS (in guinea pigs; Furman et al., 2013), there is only limited evidence that this occurs also in the case of age-related CS (gerbil data show this only  >  6 kHz; Schmiedt et al., 1996). While phenomenological profiles of ABR wave I responses appear similar for noise- and age-related CS in mice (Sergeyenko et al., 2013), the picture is more complex for FFR responses. Relative to controls, FFR growth functions with level appear shallower for mice with noise-induced synaptopathy (Shaheen et al., 2015), but for mice with age-related synaptopathy the functions seem to have simply a downward offset at all levels, that does not change their overall shape (only at equal sensation levels the functions are shallower than for controls; Parthasarathy and Kujawa, 2018). Moreover, contrary to the predictions of some models (Bharadwaj et al., 2014), in mice with age-related CS, FFR growth functions with modulation depth had similar shapes across the age range (Parthasarathy and Kujawa, 2018). Finally, a major hypothesized pathway leading to a preferential L/M-SR fiber involvement in CS, glutamate excitotoxicity (Liberman and Kujawa, 2017), does not easily apply to the case of age-related CS.

Overall, given the currently available evidence, it cannot be excluded that age-related CS may have a different profile than noise-induced CS *re* the proportion of the affected types of auditory nerve fibers in rodents or in humans. If this is the case, some of the age-related electrophysiological changes observed in the current study, such as the ABR wave I reduction at low stimulus levels with HP noise, and the general FFR ENV SNR reductions at the low carrier frequency could reflect CS that is not specific to L/M-SR fibers. However, as mentioned before, they could also reflect sensorineural deficits other than CS: if CS effects are not level specific, it becomes difficult to distinguish them from other sensorineural deficits on the basis of the electrophysiological measures employed in the current study.

## Declaration of Competing Interest

The authors declare that they have no conflict of interest.

## Appendix A Supplementary materials

Supplementary Data S1 Supplementary Raw Research Data. This is open data under the CC BY license http://creativecommons.org/licenses/by/4.0/ Supplementary Data S1

## Footnotes

1 The high correlation between age and PTA${}_{4-12}$ may suggest a multicollinearity issue for the ABR model in quiet which included both these predictors. However, (i) the variance inflation factors (VIFs) for age (3.44), and PTA${}_{4-12}$ (4.06) were moderate, and serious multicollinearity issues generally occur for VIFs  >  10 (Hair et al., 2014); (ii) the use of shrinkage priors reduces the instability of coefficients caused by multicollinearity (Chatterjee, Hadi, 2006, Hair, Black, Babin, Anderson, 2014). In any case, multicollinearity does not lead to biased estimates of the regression coefficients; the major negative consequence of multicollinearity is increased uncertainty in the estimates of the affected regression coefficients (Voss, 2005). This uncertainty is reflected in the width of their CIs (Kruschke, 2014).

2 The montage-specific results, which are presented in the SM, hint at the possibility that these across-CF differences may interact with electrode montage, because a trend for age-related reductions for the 2-kHz CF was only present for the HF-LMST montage. Trends for this montage at 2 kHz appear somewhat different from the other montages also with respect to the effect of PTA${}_{1-2},$ further suggesting that the FFR ENV at the HF-LMST montage may reflect different generators than the other montages. Unfortunately, our study, and those of Leigh-Paffenroth and Fowler (2006), and Grose et al. (2009), all used different montages and/or mode of presentation (monaural vs diotic), so it is difficult to draw general conclusions about the role of electrode montage on these across-CF differences.
