# Supplementary material for: Effects of age on electrophysiological measures of cochlear synaptopathy in humans
Source: Hear Res. 2020 Oct;396:108068. doi: 10.1016/j.heares.2020.108068 (PMC7593961; doi:10.1016/j.heares.2020.108068)
Supplement: Supplementary Data S1 — Supplementary Raw Research Data. This is open data under the CC BY license http://creativecommons.org/licenses/by/4.0/ [file mmc1.pdf]

# Supplementary Materials for "Effects of Age on Electrophysiological Measures of Cochlear Synaptopathy in Humans"

## Contents


**1 Supplementary figures referenced in the main manuscript** **2**

**2 Supplementary methods** **11**
2.1 Choice of ABR montages . . . 11
2.2 ABR waves peak-picking algorithm . . . 11
2.3 Noise exposure . . . 13
2.4 Audiometric thresholds . . . 13

**3 Statistical models and results** **14**
3.1 Bayesian correlation model . . . 14
3.2 Mixed effect multiple regression models . . . 14

**4 Supplementary results** **28**
4.1 ABR wave amplitudes for the HF-IERL montage . . . 28
4.2 ABR wave latencies for the HF-IERL montage . . . 33
4.3 FFR ENV SNR results for each montage . . . 38
4.4 FFR TFS SNR results for each montage . . . 45
4.5 FFR ENV latency results for each montage . . . 48

# 1 Supplementary figures referenced in the main manuscript

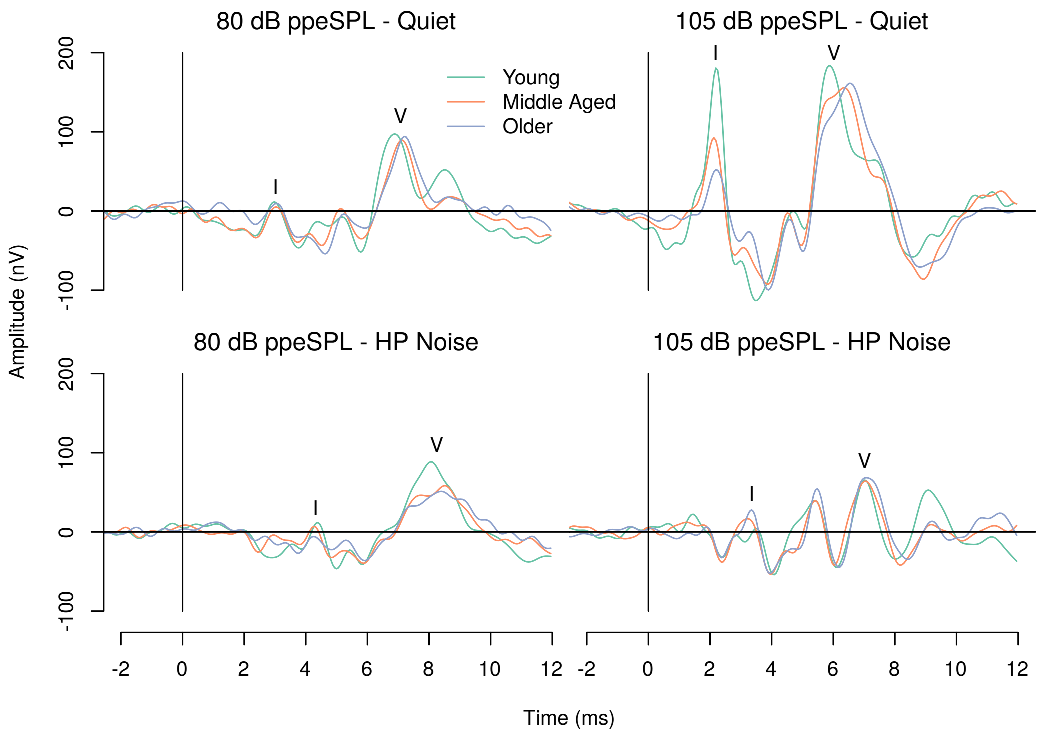


Figure S1: ABR grand averages with the HF-ITPR montage for each experimental condition as a function of age group. It should be noted that although the grand averages are shown as a function of age group for illustration purposes here, the statistical analyses used age as a continuous rather than nominal variable.

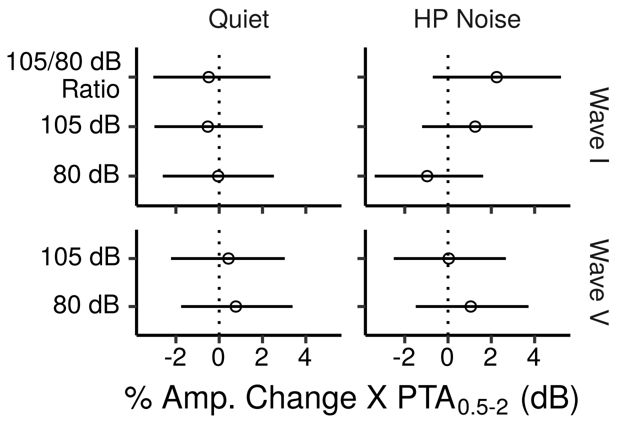


Figure S2: Posterior medians (circles) and 99% credibility intervals for the effects of $PTA_{0.5-2}$ on ABR wave I and V amplitudes estimated by the Bayesian multiple regression models.

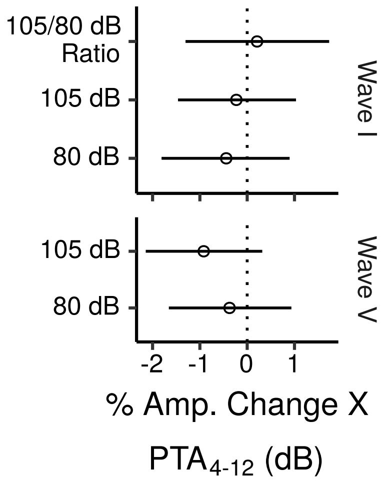


Figure S3: Posterior medians (circles) and 99% credibility intervals for the effects of $PTA_{4-12}$ on ABR wave I and V amplitudes estimated by the Bayesian multiple regression model.

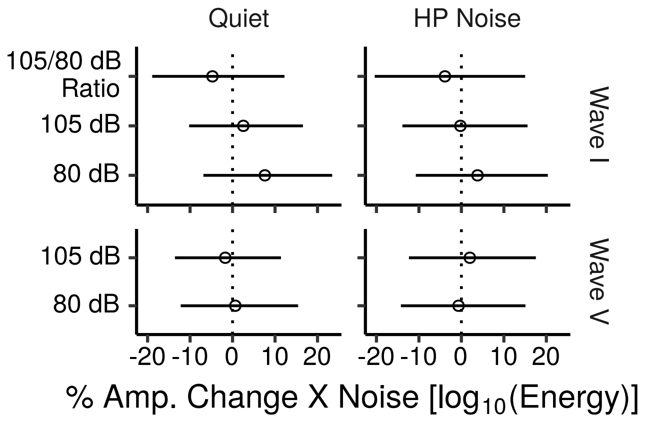


Figure S4: Posterior medians (circles) and 99% credibility intervals for the effects of $\log_{10}$TCNE on ABR wave I and V amplitudes estimated by the Bayesian multiple regression models.

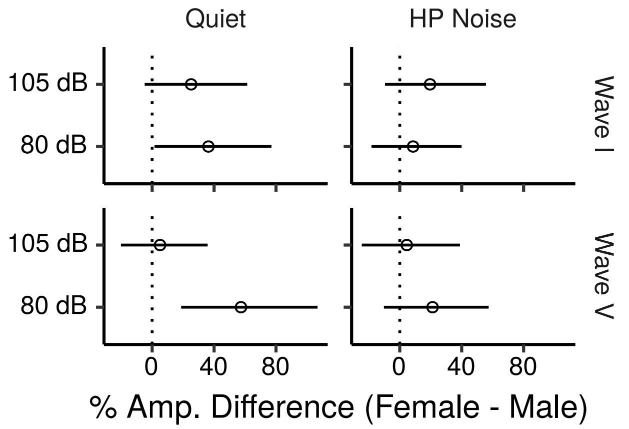


Figure S5: Posterior medians (circles) and 99% credibility intervals for the differences in ABR wave I and V amplitudes between females and males estimated by the Bayesian multiple regression models. The multiple regression models did not include montage by sex interactions, only the overall effects of sex across montages were estimated, hence the plot does not show montage specific sex effects.

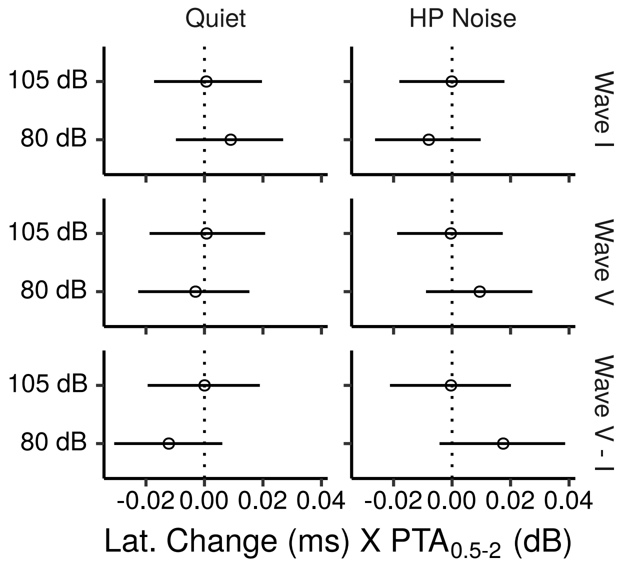


Figure S6: Posterior medians (circles) and 99% credibility intervals for the effects of $PTA_{0.5-2}$ on ABR wave I and V latencies estimated by the Bayesian multiple regression models. The bottom row shows the effects for the wave I–V interpeak latencies.

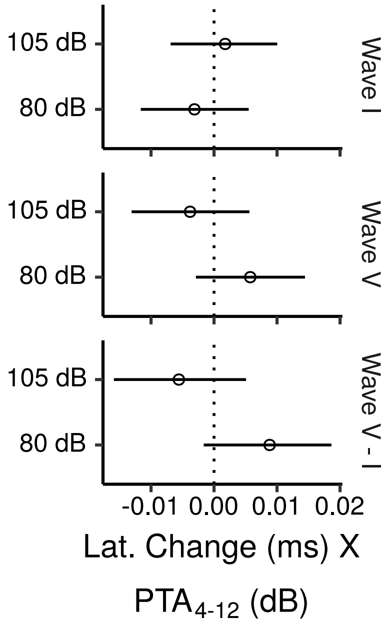


Figure S7: Posterior medians (circles) and 99% credibility intervals for the effects of $PTA_{4-12}$ on ABR wave I and V latencies estimated by the Bayesian multiple regression model. The bottom row shows the effects for the wave I–V interpeak latencies.

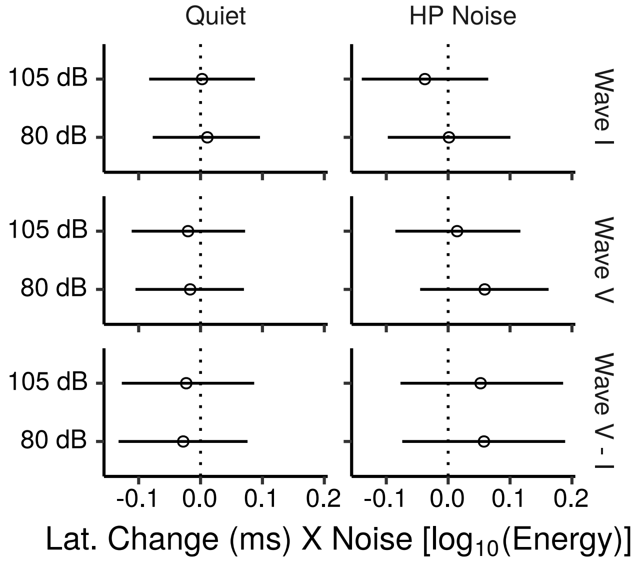


Figure S8: Posterior medians (circles) and 99% credibility intervals for the effects of $\log_{10}$TCNE on ABR wave I and V latencies estimated by the Bayesian multiple regression models. The bottom row shows the effects for the wave I–V interpeak latencies.

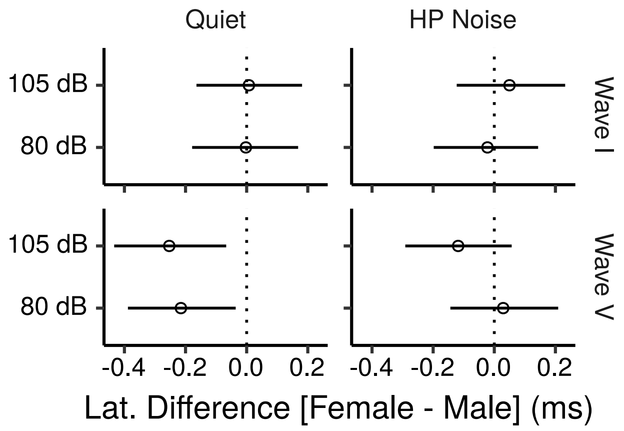


Figure S9: Posterior medians (circles) and 99% credibility intervals for the differences in ABR wave I and V latencies between females and males estimated by the Bayesian multiple regression models. The multiple regression models did not include montage by sex interactions, only the overall effects of sex across montages were estimated, hence the plot does not show montage specific sex effects.

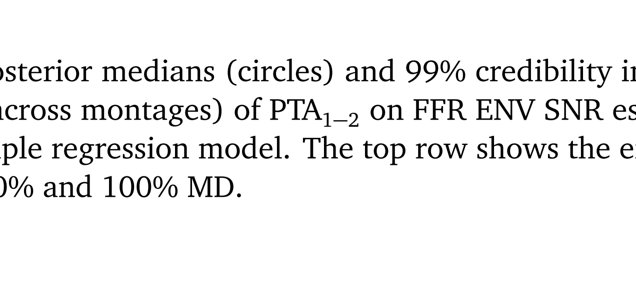


Figure S10: Posterior medians (circles) and 99% credibility intervals for the main effects (across montages) of $PTA_{1-2}$ on FFR ENV SNR estimated by the Bayesian multiple regression model. The top row shows the effect difference between the 70% and 100% MD.

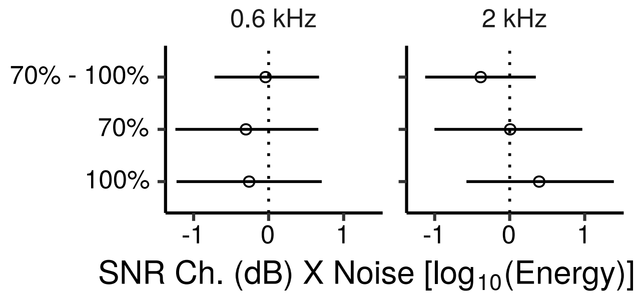


Figure S11: Posterior medians (circles) and 99% credibility intervals for the main effects (across montages) of $\log_{10}$TCNE on FFR ENV SNR estimated by the Bayesian multiple regression model. The top row shows the effect difference between the 70% and 100% MD.

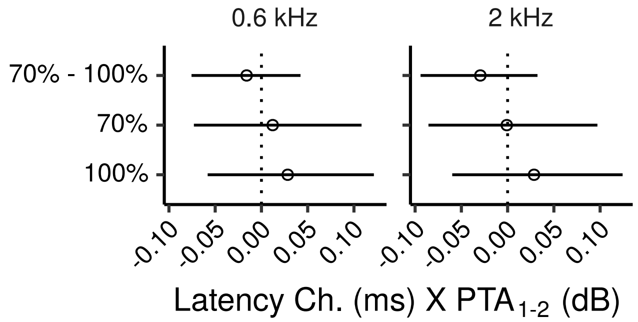


Figure S12: Posterior medians (circles) and 99% credibility intervals for the main effects (across montages) of $PTA_{1-2}$ on FFR ENV latency estimated by the Bayesian multiple regression model. The top row shows the effect difference between the 70% and 100% MD.

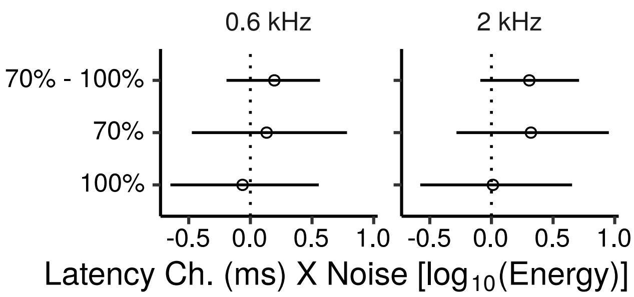


Figure S13: Posterior medians (circles) and 99% credibility intervals for the main effects (across montages) of $\log_{10}$TCNE on FFR ENV latency estimated by the Bayesian multiple regression model. The top row shows the effect difference between the 70% and 100% MD.

# 2 Supplementary methods

## 2.1 Choice of ABR montages

The data from the HF-IERL, and HF-ITPR montages were retained for the statistical analyses, while the IMST referenced data were not analyzed further for the following reasons: i) the average root-mean-square (RMS) amplitude during the pre-stimulus baseline window, indicative of noise level, was $\sim 1.5$ dB higher for the IMST referenced data than for the other two montages, that had similar baseline RMS amplitudes; ii) the ratio of the average ABR wave I amplitude to the amplitude of a dummy wave estimated with the same criteria as wave I during the pre-stimulus baseline window (Prendergast et al., 2017) was $> 1$ dB lower for the HF-IMST montage compared to the other two montages (HF-IERL: 4.42 dB; HF-ITPR: 5.1 dB; HF-IMST: 3.38 dB) iii) the HF-IMST data were often contaminated by the postauricular muscle reflex (PAM). Although the PAM triggered by the click falls outside the time-window of ABR waves I and V, the PAM response to the onset of the noise preceding the click could affect responses in this time window. The PAM to the onset of the noise should cancel out in the long run through averaging, due to its random start time with respect to the averaging window start time. However, given the large size of the PAM it is possible that the cancellation was not complete; iv) previous studies indicate that tiptrode-referenced ABRs provide larger, and slightly more reliable wave I amplitudes compared to mastoid-referenced ABRs (Bauch and Olsen, 1990; Prendergast et al., 2018).

The metrics described above suggest that the HF-ITPR montage may provide somewhat better measurements of wave I over the HF-IERL montage, but the differences between the two montages are likely small. For this reason, and because multilevel models generally provide better parameter estimates compared to separate analyses for each level of a given factor (Gelman, 2006; Gelman and Hill, 2007), the HF-ITPR and HF-IERL ABR data were modeled jointly in the statistical analyses.

## 2.2 ABR waves peak-picking algorithm

ABR peaks were first identified on the grand-average waveforms using a semi-automatic peak-picking procedure similar to that proposed by Bradley and Wilson (2004). The approximate latency for each wave was first identified visually on the grand average waveforms separately for each condition. The

grand-average peaks for each wave were then defined as the highest local maxima within a tolerance window of ±0.63-ms for wave V, and ±0.51-ms for wave I of the visually identified peak latencies.

The wave peaks were then searched in the individual subject waveforms within a search window centered at the grand-average peak latencies, and with bounds of ±0.51 ms of the grand-average peak latency for wave I, and of ±0.84 ms for wave V. These bounds correspond respectively to ±3, and ±4 standard deviations of the ABR wave latencies reported by Issa and Ross (1995) for wave I, and for wave V. Peaks were identified by selecting the highest local maximum in the search window. Troughs were identified by selecting the lowest local minimum in a search window going from 0.25 to 1.5 ms for wave I, and from 0.25 to 2 ms for wave V, from the estimated peak latency. Wave amplitudes were measured from peak to trough. If no local maxima were present in the peak search window the peak amplitude was estimated by the highest absolute point in the search window (this point was also used to set the search window for the following trough). If no local minima were present in the trough search window, the trough amplitude was estimated by the inflection point with the shallowest slope in the trough search window, if present. If no inflection points were present the trough amplitude was estimated by the lowest absolute point in the trough search window.

It should be noted that because of the constraint that the trough latency is at least 0.25 ms after the peak latency there is no guarantee that either the local or the absolute minimum found in the trough search window will have a lower amplitude than the peak found in the peak search window. Therefore the algorithm could fail to find positive peak-trough amplitudes. This usually occurred for noisy waveforms. In these cases the peak-trough amplitude was set as missing. A censored analysis, described in the section on statistical methods was then used to deal with these missing data.

Latencies for peaks that could not be identified using the largest local maximum were set as missing. The rationale for this is that while the largest absolute maximum can provide a reasonable estimate of the peak amplitude when no local maxima are present, it does not necessarily provide a good estimate of the peak latency. Peak latencies for peaks with an amplitude smaller than 100 nV were also considered unreliable and set as missing.

The noise floor was calculated by applying the same algorithm used to find wave I to the pre-stimulus baseline, on a time window centered at -2.1 ms.

## 2.3 Noise exposure

Lifetime noise exposure was estimated via the structured interview developed by Lutman et al. (2008). The interview covers both occupational and recreational noise exposures. For each of these two categories participants were asked to recall up to five activities with the greatest amount of noise exposure in their lifetime, and with levels exceeding a threshold of 85 dBA. Noise levels were estimated mostly using a speech communication difficulty table that listed approximate noise levels as a function of vocal effort required for communication. For each activity participants were asked to estimate the duration and frequency of occurrence, and the cumulative noise exposure for the activity ($U$) was calculated by $U = 10^{(L-A-90)/10} \cdot Y \cdot W \cdot D \cdot H/2080$ where $L$ is estimated noise exposure level in dBA, $A$ is hearing protection in dB, $Y$ is years of exposure, $W$ is weeks of exposure per year, $D$ is days of exposure per week, $H$ is hours of exposure per day, and 2080 is the number of hours in a working year. One unit of noise exposure so calculated corresponds to an eight hour daily exposure, for five days a week, for 52 weeks, for a year, to a noise level of 90 dBA. The cumulative noise exposure was summed across all activities to estimate the total cumulative noise exposure (TCNE). For the analyses the TCNE was log-transformed using base 10, so that a unit difference in the $\log_{10}$-transformed TCNE corresponds to a tenfold difference in noise exposure energy.

## 2.4 Audiometric thresholds

The tones had a duration of 200 ms, including 10-ms raised-cosine onset and offset ramps. Thresholds were measured with a two-interval two-alternative forced-choice task. The presentation level of each tone was varied adaptively using a two-down one-up transformed up-down procedure tracking the 70.7% correct point on the psychometric function (Levitt, 1971) to determine its detection threshold. On each trial the tone was randomly presented during one of two observation intervals marked by flashing lights on the computer screen, and separated by a 500-ms silent interval. Participants were asked to indicate the interval in which the sound occurred by pressing the corresponding button on a numeric keypad. Feedback was provided at the end of each trial by means of a colored light on the computer screen.

A single block of trials was run for each combination of ear and frequency (in random order). Each block was terminated after 16 turnpoints of the adaptive track. The level was varied in 4-dB steps for the first four turnpoints, and by 2

dB for the remaining turnpoints. The threshold was estimated as the average of the last 12 turnpoints.

The pure tones were synthesized in Python (Python Software Foundation, Delaware, United States) with a sampling rate of 48 kHz, and 32-bit depth, were played through a E-MU 0204 USB sound card (E-MU Systems, Scotts Valley, U.S.A.), and presented via Sennheiser HDA300 headphones (Sennheiser electronic GmbH & Co. KG, Hanover, Germany). Testing took place in double-walled IAC (IAC Acoustics, Winchester, UK) soundproof booths.

# 3 Statistical models and results

All analyses were performed using Bayesian models implemented by Markov Chain Monte Carlo (MCMC) simulations using JAGS (Plummer, 2003) and R (R Core Team, 2020). For all MCMC simulations the chains for the main parameters of interest were monitored for convergence using trace plots, and where available the Gelman-Rubin statistics. The chains were also monitored for autocorrelation to ensure an effective sample size of at least $\simeq 10,000$ samples for the main parameters of interest.

## 3.1 Bayesian correlation model

The Bayesian model to estimate the correlations among predictor variables was based on the model of Lee and Wagenmakers (2014, chap. 5) but used vague uniform priors for estimating the standard deviations of the variables instead of inverse-square-root-gamma priors.

## 3.2 Mixed effect multiple regression models

The data were analyzed using robust mixed-effect multiple regression models which included both categorical and continuous predictors, as well as random effects of subjects. Robust regression uses a Student's $t$ distribution instead of a Normal distribution for describing residuals, minimizing the potential influence of outliers on the estimated regression coefficients (Kruschke, 2014).

For categorical predictors an unweighted effect coding scheme was used (Aiken et al., 1991). Continuous variables were standardized using the Friedrich method (Friedrich, 1982; Aiken et al., 1991) before being entered into the analyses. Unstandardized coefficients corresponding to those resulting from an

analysis of the mean-centered variables can be obtained by scaling using the appropriate standard deviation terms (Aiken et al., 1991). The priors for the slope coefficients in the models were set differently for coefficients that were of main interest in the analysis, and coefficients that were expected to affect the dependent variable, but were not of great analytical interest, such as the effect of stimulus level on ABR amplitude. For the latter effects, the priors were very broad on the scale of the data. Shrinkage priors were used for the former: the standardized coefficients were described by a *t* distribution centered at zero, with 1 degree of freedom, and scale parameter set to 0.1. This prior assumes that the standardized slope coefficients should be generally close to zero, where the narrow peak of the *t* distribution is located, reflecting a belief that effect sizes will be generally small. However, owing to its heavy tails the *t* prior can accommodate coefficients much larger than zero if the likelihood provides clear evidence for this (Kruschke, 2014).

The interpretation of the standardized slope coefficients, and hence of the priors set on them, differs for continuous and categorical variables. For continuous variables the standardized slope coefficient is the change of the dependent variable in standard deviation (sd) units, for a 1-sd change of the dependent variable. Categorical variables were not standardized, and the coefficients represent the shift in the value of the dependent variable (which was still set in sd units in our models) for the categorical level coded as 1, from the the unweighted grand mean of the dependent variable of all the levels. The model code is available at https://doi.org/10.17605/OSF.IO/S3BD9.

Tables S1, and S2 indicate the dummy codes used to encode categorical variables through an unweighted effect coding scheme. Tables S3, S4, S5, S6, S7, S8, and S9 list all the terms included in each statistical model (excluding the random effect of participant). The first column indicates the variable to which each coefficient refers (abbreviated as previously defined in the main text of the manuscript or as indicated in Tables S1 and S2). The second column indicates the type of variable (continuous, categorical, or interaction). The third column indicates (for all the terms except the intercept) the scale parameter of the 1-degree-of-freedom *t* distribution used as a prior for the standardized slope coefficient; for the intercept term this column indicates the standard deviation of the zero-centered normal distribution used as a prior for the intercept. The fourth column indicates the same quantity as the third column, but in unstandardized mean-centered units. The fifth column indicates the median of the posterior distribution in unstandardized mean-centered units. The sixth column indicates the 99% CI for the coefficients in unstandardized

mean-centered units.

| Abbreviation | Variable | Dummy codes |
|---|---|---|
| A | Age decade | |
| L | Level | -1=80 dB ppeSPL, 1=105 dB ppeSPL |
| W | Wave | -1=I, 1=V |
| M | Montage | -1=HF-IERL, 1=HF-ITPR |
| S | Sex | -1=Male, 1=Female |

Table S1: Abbreviated variable names for the ABR models. The third column lists the dummy codes used for each categorical variable.

| Abbreviation | Variable | Dummy codes |
|---|---|---|
| A | Age decade | |
| $\sqrt[3]{\text{Y}}$ | Cube root music years | |
| CF | Carrier frequency | -1=0.6 kHz, 1=2 kHz |
| MD | Modulation depth | -1=70%, 1=100% |
| M1 | Montage 1 | -1=HF-LTPR, 1=HF-C7<br>0=HF-LERL or HF-LMST |
| M2 | Montage 2 | -1=HF-LTPR, 1=HF-LERL<br>0=HF-C7 or HF-LMST |
| M3 | Montage 3 | -1=HF-LTPR, 1=HF-LMST<br>0=HF-C7 or HF-LERL |

Table S2: Abbreviated variable names for the FFR models. M1, M2 and M3 are the coefficients for the three dummy variables needed to encode the four levels of the montage factor. The third column lists the dummy codes used for each categorical variable.

To give a sense of the prior distribution Fig. S14 plots $t$ distributions with the same mean and degrees of freedom as the priors used in the current study for several values of the scale parameter. In each case the prior probability is highest for values around zero; while it is sharply centered around zero for small scale values, it becomes more diffuse as the scale value increases. Even when the scale value is relatively small, due to its heavy tails the $t$ distributions

can accommodate coefficients much larger than zero if the likelihood provides clear evidence for this. For a more in depth overview of $t$ priors see Kruschke (2014).

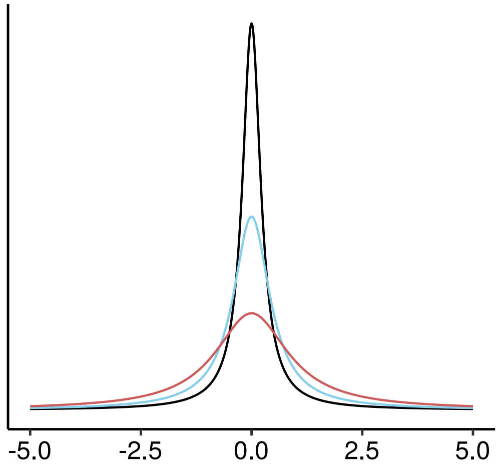


Figure S14: Density functions of $t$ distributions with 1 degree of freedom centered at zero, and with scale parameter set to 0.25 (black), 0.5 (light blue), or 1 (red).

The models for the ABR wave amplitude (Table S4) and latency (Table S6) in quiet had two predictors (and relative interaction terms) for audiometric thresholds instead of a single one: One predictor for low-frequency audiometric thresholds ($\text{PTA}_{0.5-2}$), and one for high-frequency audiometric thresholds ($\text{PTA}_{4-12}$). The choice of these two predictors was motivated by several factors: i) age-related audiometric losses typically are not flat across the frequency range, but more pronounced at frequencies $\gtrsim 2$ kHz ii) with broadband stimulation the contribution of lower and higher cochlear frequency regions to ABR wave amplitudes is level dependent (Don and Eggermont, 1978; Eggermont and Don, 1980), with a breakpoint occurring roughly around 2 kHz, hence an interaction term between frequency region and stimulus level is needed to capture the effects of audiometric loss on ABR wave amplitudes at different levels.

Residuals plus component plots (Ezekiel, 1924; Chatterjee and Hadi, 2006; Fox, 2016) were used to check that relations between the dependent variable and each predictor (with the effects of other predictors partialed out) were approximately linear.

Table S3: Model terms and coefficients for the ABR amplitude in HP noise model.

| **Coefficient** | **Type** | **Prior scale** $z$ | **Prior scale** | **Posterior Median** | **99% CI** |
|---|---|---|---|---|---|
| Intercept | | 2.000 | 1.286 | 4.673 | 4.588–4.756 |
| A | Cont. | 0.100 | 0.036 | -0.057 | -0.104– -0.011 |
| $PTA_{0.5-2}$ | Cont. | 0.100 | 0.011 | 0.003 | -0.014–0.021 |
| $log_{10}TCNE$ | Cont. | 0.100 | 0.077 | 0.017 | -0.067–0.106 |
| L | Cat. | 10.000 | 6.430 | 0.138 | 0.085–0.189 |
| W | Cat. | 10.000 | 6.430 | 0.195 | 0.144–0.247 |
| M | Cat. | 10.000 | 6.430 | 0.015 | -0.023–0.054 |
| S | Cat. | 10.000 | 6.430 | 0.062 | -0.048–0.167 |
| LxW | Int. | 0.100 | 0.064 | 0.050 | -0.002–0.102 |
| LxM | Int. | 0.100 | 0.064 | 0.029 | -0.009–0.066 |
| LxWxM | Int. | 0.100 | 0.064 | -0.022 | -0.06–0.015 |
| LxS | Int. | 0.100 | 0.064 | -0.006 | -0.058–0.046 |
| LxWxS | Int. | 0.100 | 0.064 | -0.030 | -0.084–0.02 |
| LxA | Int. | 0.100 | 0.036 | 0.060 | 0.036–0.085 |
| $LxPTA_{0.5-2}$ | Int. | 0.100 | 0.011 | 0.002 | -0.006–0.009 |
| $Lxlog_{10}TCNE$ | Int. | 0.100 | 0.077 | 0.010 | -0.037–0.056 |
| LxWxA | Int. | 0.100 | 0.036 | 0.017 | -0.005–0.04 |
| $LxWxPTA_{0.5-2}$ | Int. | 0.100 | 0.011 | -0.005 | -0.013–0.003 |
| $LxWxlog_{10}TCNE$ | Int. | 0.100 | 0.077 | 0.024 | -0.022–0.072 |
| WxM | Int. | 0.100 | 0.064 | -0.051 | -0.091– -0.013 |
| WxS | Int. | 0.100 | 0.064 | -0.003 | -0.055–0.047 |
| WxA | Int. | 0.100 | 0.036 | -0.004 | -0.026–0.019 |
| $WxPTA_{0.5-2}$ | Int. | 0.100 | 0.011 | 0.003 | -0.004–0.011 |
| $Wxlog_{10}TCNE$ | Int. | 0.100 | 0.077 | -0.018 | -0.065–0.029 |
| MxA | Int. | 0.100 | 0.036 | -0.014 | -0.035–0.01 |
| $MxPTA_{0.5-2}$ | Int. | 0.100 | 0.011 | 0.001 | -0.007–0.008 |
| $Mxlog_{10}TCNE$ | Int. | 0.100 | 0.077 | -0.005 | -0.05–0.04 |
| LxMxA | Int. | 0.100 | 0.036 | 0.010 | -0.012–0.032 |
| $LxMxPTA_{0.5-2}$ | Int. | 0.100 | 0.011 | 0.001 | -0.006–0.009 |
| $LxMxlog_{10}TCNE$ | Int. | 0.100 | 0.077 | -0.013 | -0.057–0.032 |
| WxMxA | Int. | 0.100 | 0.036 | 0.008 | -0.014–0.031 |
| $WxMxPTA_{0.5-2}$ | Int. | 0.100 | 0.011 | -0.001 | -0.009–0.006 |
| $WxMxlog_{10}TCNE$ | Int. | 0.100 | 0.077 | 0.013 | -0.032–0.058 |

| | | | | | |
|---|---|---|---|---|---|
| LxWxMxA | Int. | 0.100 | 0.036 | 0.002 | -0.02–0.024 |
| LxWxMxPTA$_{0.5-2}$ | Int. | 0.100 | 0.011 | -0.003 | -0.011–0.004 |
| LxWxMxlog$_{10}$TCNE | Int. | 0.100 | 0.077 | -0.008 | -0.053–0.037 |
| SxPTA$_{0.5-2}$ | Int. | 0.100 | 0.011 | 0.001 | -0.015–0.018 |

Table S4: Model terms and coefficients for the ABR amplitude in quiet model.

| **Coefficient** | **Type** | **Prior scale $z$** | **Prior scale** | **Posterior Median** | **99% CI** |
|---|---|---|---|---|---|
| Intercept | | 2.000 | 1.451 | 5.013 | 4.924–5.097 |
| A | Cont. | 0.100 | 0.041 | -0.037 | -0.109–0.029 |
| PTA$_{0.5-2}$ | Cont. | 0.100 | 0.013 | 0.004 | -0.013–0.024 |
| PTA$_{4-12}$ | Cont. | 0.100 | 0.004 | -0.006 | -0.014–0.002 |
| log$_{10}$TCNE | Cont. | 0.100 | 0.087 | 0.020 | -0.063–0.104 |
| L | Cat. | 10.000 | 7.255 | 0.377 | 0.334–0.422 |
| W | Cat. | 10.000 | 7.255 | 0.179 | 0.136–0.225 |
| M | Cat. | 10.000 | 7.255 | 0.021 | -0.013–0.054 |
| S | Cat. | 10.000 | 7.255 | 0.130 | 0.023–0.24 |
| LxW | Int. | 0.100 | 0.073 | -0.094 | -0.14– -0.047 |
| LxM | Int. | 0.100 | 0.073 | 0.008 | -0.024–0.041 |
| LxWxM | Int. | 0.100 | 0.073 | -0.027 | -0.061–0.005 |
| LxS | Int. | 0.100 | 0.073 | -0.061 | -0.108– -0.014 |
| LxWxS | Int. | 0.100 | 0.073 | -0.040 | -0.09–0.006 |
| LxA | Int. | 0.100 | 0.041 | -0.044 | -0.079– -0.011 |
| LxPTA$_{0.5-2}$ | Int. | 0.100 | 0.013 | -0.003 | -0.01–0.005 |
| LxPTA$_{4-12}$ | Int. | 0.100 | 0.004 | 0.000 | -0.004–0.004 |
| Lxlog$_{10}$TCNE | Int. | 0.100 | 0.087 | -0.021 | -0.061–0.021 |
| LxWxA | Int. | 0.100 | 0.041 | 0.031 | -0.002–0.065 |
| LxWxPTA$_{0.5-2}$ | Int. | 0.100 | 0.013 | -0.002 | -0.009–0.006 |
| LxWxPTA$_{4-12}$ | Int. | 0.100 | 0.004 | -0.000 | -0.004–0.003 |
| LxWxlog$_{10}$TCNE | Int. | 0.100 | 0.087 | 0.001 | -0.04–0.042 |
| WxM | Int. | 0.100 | 0.073 | -0.049 | -0.083– -0.017 |
| WxS | Int. | 0.100 | 0.073 | -0.004 | -0.049–0.042 |
| WxA | Int. | 0.100 | 0.041 | 0.042 | 0.008–0.077 |
| WxPTA$_{0.5-2}$ | Int. | 0.100 | 0.013 | 0.004 | -0.003–0.011 |
| WxPTA$_{4-12}$ | Int. | 0.100 | 0.004 | -0.000 | -0.004–0.003 |

| | | | | | |
|---|---|---|---|---|---|
| Wx$\log_{10}$TCNE | Int. | 0.100 | 0.087 | -0.029 | -0.069–0.012 |
| MxA | Int. | 0.100 | 0.041 | -0.021 | -0.054–0.01 |
| Mx$PTA_{0.5-2}$ | Int. | 0.100 | 0.013 | -0.003 | -0.01–0.003 |
| Mx$PTA_{4-12}$ | Int. | 0.100 | 0.004 | 0.001 | -0.002–0.005 |
| Mx$\log_{10}$TCNE | Int. | 0.100 | 0.087 | 0.001 | -0.037–0.04 |
| LxMxA | Int. | 0.100 | 0.041 | 0.009 | -0.023–0.041 |
| LxMx$PTA_{0.5-2}$ | Int. | 0.100 | 0.013 | 0.001 | -0.006–0.008 |
| LxMx$PTA_{4-12}$ | Int. | 0.100 | 0.004 | -0.001 | -0.005–0.003 |
| LxMx$\log_{10}$TCNE | Int. | 0.100 | 0.087 | 0.003 | -0.037–0.041 |
| WxMxA | Int. | 0.100 | 0.041 | 0.015 | -0.017–0.047 |
| WxMx$PTA_{0.5-2}$ | Int. | 0.100 | 0.013 | 0.001 | -0.006–0.007 |
| WxMx$PTA_{4-12}$ | Int. | 0.100 | 0.004 | -0.001 | -0.005–0.002 |
| WxMx$\log_{10}$TCNE | Int. | 0.100 | 0.087 | 0.001 | -0.038–0.04 |
| LxWxMxA | Int. | 0.100 | 0.041 | 0.004 | -0.027–0.036 |
| LxWxMx$PTA_{0.5-2}$ | Int. | 0.100 | 0.013 | 0.002 | -0.004–0.009 |
| LxWxMx$PTA_{4-12}$ | Int. | 0.100 | 0.004 | -0.001 | -0.005–0.002 |
| LxWxMx$\log_{10}$TCNE | Int. | 0.100 | 0.087 | 0.005 | -0.035–0.043 |
| Sx$PTA_{0.5-2}$ | Int. | 0.100 | 0.013 | 0.002 | -0.015–0.021 |
| Sx$PTA_{4-12}$ | Int. | 0.100 | 0.004 | -0.001 | -0.007–0.004 |

Table S5: Model terms and coefficients for the ABR latency in HP noise model.

| **Coefficient** | **Type** | **Prior scale $z$** | **Prior scale** | **Posterior Median** | **99% CI** |
|---|---|---|---|---|---|
| Intercept | | 2.000 | 4.024 | 5.718 | 5.666–5.77 |
| A | Cont. | 0.100 | 0.114 | 0.008 | -0.017–0.035 |
| $PTA_{0.5-2}$ | Cont. | 0.100 | 0.036 | -0.001 | -0.012–0.011 |
| $\log_{10}$TCNE | Cont. | 0.100 | 0.240 | 0.009 | -0.045–0.062 |
| L | Cat. | 10.000 | 20.118 | -0.528 | -0.563– -0.494 |
| W | Cat. | 10.000 | 20.118 | 1.924 | 1.889–1.958 |
| M | Cat. | 10.000 | 20.118 | 0.025 | -0.003–0.052 |
| S | Cat. | 10.000 | 20.118 | -0.008 | -0.072–0.052 |
| LxW | Int. | 0.100 | 0.201 | -0.032 | -0.066–0.001 |
| LxM | Int. | 0.100 | 0.201 | 0.011 | -0.017–0.037 |
| LxWxM | Int. | 0.100 | 0.201 | -0.005 | -0.031–0.022 |
| LxS | Int. | 0.100 | 0.201 | -0.009 | -0.046–0.026 |
| LxWxS | Int. | 0.100 | 0.201 | -0.027 | -0.064–0.008 |

| | | | | | |
|---|---|---|---|---|---|
| LxA | Int. | 0.100 | 0.114 | -0.002 | -0.018–0.015 |
| LxPTA$_{0.5-2}$ | Int. | 0.100 | 0.036 | 0.001 | -0.005–0.006 |
| Lxlog$_{10}$TCNE | Int. | 0.100 | 0.240 | -0.023 | -0.058–0.01 |
| LxWxA | Int. | 0.100 | 0.114 | 0.009 | -0.008–0.025 |
| LxWxPTA$_{0.5-2}$ | Int. | 0.100 | 0.036 | -0.004 | -0.009–0.002 |
| LxWxlog$_{10}$TCNE | Int. | 0.100 | 0.240 | -0.007 | -0.04–0.027 |
| WxM | Int. | 0.100 | 0.201 | -0.013 | -0.04–0.014 |
| WxS | Int. | 0.100 | 0.201 | -0.015 | -0.051–0.02 |
| WxA | Int. | 0.100 | 0.114 | 0.018 | 0.001–0.034 |
| WxPTA$_{0.5-2}$ | Int. | 0.100 | 0.036 | 0.001 | -0.004–0.007 |
| Wxlog$_{10}$TCNE | Int. | 0.100 | 0.240 | 0.015 | -0.019–0.047 |
| MxA | Int. | 0.100 | 0.114 | 0.003 | -0.014–0.019 |
| MxPTA$_{0.5-2}$ | Int. | 0.100 | 0.036 | 0.001 | -0.004–0.006 |
| Mxlog$_{10}$TCNE | Int. | 0.100 | 0.240 | -0.000 | -0.032–0.033 |
| LxMxA | Int. | 0.100 | 0.114 | 0.005 | -0.012–0.021 |
| LxMxPTA$_{0.5-2}$ | Int. | 0.100 | 0.036 | -0.001 | -0.006–0.004 |
| LxMxlog$_{10}$TCNE | Int. | 0.100 | 0.240 | 0.002 | -0.03–0.035 |
| WxMxA | Int. | 0.100 | 0.114 | -0.003 | -0.02–0.013 |
| WxMxPTA$_{0.5-2}$ | Int. | 0.100 | 0.036 | 0.003 | -0.002–0.008 |
| WxMxlog$_{10}$TCNE | Int. | 0.100 | 0.240 | 0.013 | -0.02–0.045 |
| LxWxMxA | Int. | 0.100 | 0.114 | 0.002 | -0.014–0.019 |
| LxWxMxPTA$_{0.5-2}$ | Int. | 0.100 | 0.036 | -0.001 | -0.006–0.005 |
| LxWxMxlog$_{10}$TCNE | Int. | 0.100 | 0.240 | 0.005 | -0.027–0.038 |
| SxPTA$_{0.5-2}$ | Int. | 0.100 | 0.036 | 0.000 | -0.011–0.011 |

Table S6: Model terms and coefficients for the ABR latency in quiet model.

| **Coefficient** | **Type** | **Prior scale $z$** | **Prior scale** | **Posterior Median** | **99% CI** |
|---|---|---|---|---|---|
| Intercept | | 2.000 | 4.213 | 4.670 | 4.611–4.73 |
| A | Cont. | 0.100 | 0.119 | 0.024 | -0.021–0.069 |
| PTA$_{0.5-2}$ | Cont. | 0.100 | 0.037 | 0.001 | -0.013–0.016 |
| PTA$_{4-12}$ | Cont. | 0.100 | 0.012 | 0.000 | -0.005–0.006 |
| log$_{10}$TCNE | Cont. | 0.100 | 0.251 | -0.005 | -0.058–0.05 |
| L | Cat. | 10.000 | 21.066 | -0.446 | -0.475– -0.416 |
| W | Cat. | 10.000 | 21.066 | 2.070 | 2.041–2.098 |

| | | | | | |
|---|---|---|---|---|---|
| M | Cat. | 10.000 | 21.066 | 0.016 | -0.005–0.038 |
| S | Cat. | 10.000 | 21.066 | -0.058 | -0.13–0.012 |
| LxW | Int. | 0.100 | 0.211 | -0.016 | -0.046–0.013 |
| LxM | Int. | 0.100 | 0.211 | -0.009 | -0.03–0.013 |
| LxWxM | Int. | 0.100 | 0.211 | -0.006 | -0.027–0.016 |
| LxS | Int. | 0.100 | 0.211 | -0.004 | -0.033–0.028 |
| LxWxS | Int. | 0.100 | 0.211 | -0.006 | -0.036–0.024 |
| LxA | Int. | 0.100 | 0.119 | -0.004 | -0.026–0.019 |
| $LxPTA_{0.5-2}$ | Int. | 0.100 | 0.037 | 0.000 | -0.004–0.005 |
| $LxPTA_{4-12}$ | Int. | 0.100 | 0.012 | -0.001 | -0.004–0.001 |
| $Lxlog_{10}TCNE$ | Int. | 0.100 | 0.251 | -0.006 | -0.033–0.02 |
| LxWxA | Int. | 0.100 | 0.119 | 0.016 | -0.006–0.04 |
| $LxWxPTA_{0.5-2}$ | Int. | 0.100 | 0.037 | 0.003 | -0.002–0.007 |
| $LxWxPTA_{4-12}$ | Int. | 0.100 | 0.012 | -0.002 | -0.005–0 |
| $LxWxlog_{10}TCNE$ | Int. | 0.100 | 0.251 | 0.000 | -0.028–0.026 |
| WxM | Int. | 0.100 | 0.211 | -0.023 | -0.044–0 |
| WxS | Int. | 0.100 | 0.211 | -0.059 | -0.088– -0.028 |
| WxA | Int. | 0.100 | 0.119 | 0.027 | 0.005–0.051 |
| $WxPTA_{0.5-2}$ | Int. | 0.100 | 0.037 | -0.003 | -0.007–0.002 |
| $WxPTA_{4-12}$ | Int. | 0.100 | 0.012 | 0.002 | -0.001–0.004 |
| $Wxlog_{10}TCNE$ | Int. | 0.100 | 0.251 | -0.014 | -0.041–0.013 |
| MxA | Int. | 0.100 | 0.119 | 0.003 | -0.02–0.025 |
| $MxPTA_{0.5-2}$ | Int. | 0.100 | 0.037 | 0.001 | -0.004–0.006 |
| $MxPTA_{4-12}$ | Int. | 0.100 | 0.012 | -0.000 | -0.003–0.002 |
| $Mxlog_{10}TCNE$ | Int. | 0.100 | 0.251 | -0.001 | -0.027–0.025 |
| LxMxA | Int. | 0.100 | 0.119 | -0.004 | -0.027–0.018 |
| $LxMxPTA_{0.5-2}$ | Int. | 0.100 | 0.037 | -0.002 | -0.006–0.003 |
| $LxMxPTA_{4-12}$ | Int. | 0.100 | 0.012 | 0.000 | -0.002–0.003 |
| $LxMxlog_{10}TCNE$ | Int. | 0.100 | 0.251 | 0.003 | -0.024–0.029 |
| WxMxA | Int. | 0.100 | 0.119 | 0.007 | -0.016–0.029 |
| $WxMxPTA_{0.5-2}$ | Int. | 0.100 | 0.037 | -0.000 | -0.005–0.004 |
| $WxMxPTA_{4-12}$ | Int. | 0.100 | 0.012 | -0.001 | -0.003–0.002 |
| $WxMxlog_{10}TCNE$ | Int. | 0.100 | 0.251 | 0.001 | -0.024–0.028 |
| LxWxMxA | Int. | 0.100 | 0.119 | 0.007 | -0.015–0.03 |
| $LxWxMxPTA_{0.5-2}$ | Int. | 0.100 | 0.037 | 0.000 | -0.004–0.005 |
| $LxWxMxPTA_{4-12}$ | Int. | 0.100 | 0.012 | -0.001 | -0.004–0.001 |
| $LxWxMxlog_{10}TCNE$ | Int. | 0.100 | 0.251 | 0.001 | -0.025–0.028 |
| $SxPTA_{0.5-2}$ | Int. | 0.100 | 0.037 | -0.001 | -0.015–0.013 |

| | | | | | |
|---|---|---|---|---|---|
| $SxPTA_{4-12}$ | Int. | 0.100 | 0.012 | 0.003 | -0.001–0.007 |

Table S7: Model terms and coefficients for the FFR ENV SNR model.

| **Coefficient** | **Type** | **Prior scale** $z$ | **Prior scale** | **Posterior Median** | **99% CI** |
|---|---|---|---|---|---|
| Intercept | | 2.000 | 10.516 | 13.135 | 12.895–13.366 |
| A | Cont. | 0.100 | 0.298 | -0.329 | -0.837–0.133 |
| $PTA_{1-2}$ | Cont. | 0.100 | 0.087 | -0.036 | -0.177–0.09 |
| $\log_{10}$TCNE | Cont. | 0.100 | 0.627 | -0.041 | -0.945–0.789 |
| $\sqrt[3]{Y}$ | Cont. | 0.100 | 0.463 | 0.343 | -0.286–1.077 |
| CF | Cat. | 10.000 | 52.581 | -0.703 | -0.929– -0.484 |
| MD | Cat. | 10.000 | 52.581 | 0.835 | 0.622–1.063 |
| M1 | Cat. | 10.000 | 52.581 | 0.082 | -0.288–0.455 |
| M2 | Cat. | 10.000 | 52.581 | 0.066 | -0.305–0.448 |
| M3 | Cat. | 10.000 | 52.581 | 0.180 | -0.218–0.613 |
| CFxMD | Int. | 0.100 | 0.526 | -0.006 | -0.215–0.212 |
| CFxM1 | Int. | 0.100 | 0.526 | -0.382 | -0.743– -0.014 |
| CFxM2 | Int. | 0.100 | 0.526 | -0.452 | -0.831– -0.088 |
| CFxM3 | Int. | 0.100 | 0.526 | 1.302 | 0.878–1.714 |
| CFxA | Int. | 0.100 | 0.298 | 0.217 | 0.087–0.348 |
| $CFxPTA_{1-2}$ | Int. | 0.100 | 0.087 | 0.133 | 0.093–0.174 |
| CFx$\log_{10}$TCNE | Int. | 0.100 | 0.627 | 0.239 | -0.014–0.499 |
| MDxM1 | Int. | 0.100 | 0.526 | -0.023 | -0.36–0.321 |
| MDxM2 | Int. | 0.100 | 0.526 | 0.028 | -0.333–0.363 |
| MDxM3 | Int. | 0.100 | 0.526 | -0.083 | -0.456–0.294 |
| MDxA | Int. | 0.100 | 0.298 | 0.054 | -0.071–0.184 |
| $MDxPTA_{1-2}$ | Int. | 0.100 | 0.087 | -0.003 | -0.04–0.037 |
| MDx$\log_{10}$TCNE | Int. | 0.100 | 0.627 | 0.106 | -0.154–0.352 |
| M1xA | Int. | 0.100 | 0.298 | -0.020 | -0.228–0.19 |
| $M1xPTA_{1-2}$ | Int. | 0.100 | 0.087 | -0.015 | -0.078–0.046 |
| M1x$\log_{10}$TCNE | Int. | 0.100 | 0.627 | -0.180 | -0.591–0.236 |
| M2xA | Int. | 0.100 | 0.298 | 0.123 | -0.082–0.334 |
| $M2xPTA_{1-2}$ | Int. | 0.100 | 0.087 | 0.032 | -0.03–0.096 |
| M2x$\log_{10}$TCNE | Int. | 0.100 | 0.627 | -0.127 | -0.539–0.283 |
| M3xA | Int. | 0.100 | 0.298 | -0.215 | -0.449–0.013 |

| | | | | | |
|---|---|---|---|---|---|
| M3xPTA$_{1-2}$ | Int. | 0.100 | 0.087 | -0.076 | -0.149– -0.005 |
| M3xlog$_{10}$TCNE | Int. | 0.100 | 0.627 | 0.181 | -0.267–0.629 |
| CFxM1xA | Int. | 0.100 | 0.298 | 0.064 | -0.144–0.275 |
| CFxM2xA | Int. | 0.100 | 0.298 | 0.067 | -0.136–0.283 |
| CFxM3xA | Int. | 0.100 | 0.298 | -0.240 | -0.47– -0.009 |
| CFxM1xPTA$_{1-2}$ | Int. | 0.100 | 0.087 | -0.002 | -0.064–0.06 |
| CFxM2xPTA$_{1-2}$ | Int. | 0.100 | 0.087 | 0.019 | -0.042–0.084 |
| CFxM3xPTA$_{1-2}$ | Int. | 0.100 | 0.087 | -0.030 | -0.102–0.037 |
| CFxM1xlog$_{10}$TCNE | Int. | 0.100 | 0.627 | -0.138 | -0.551–0.277 |
| CFxM2xlog$_{10}$TCNE | Int. | 0.100 | 0.627 | -0.108 | -0.523–0.302 |
| CFxM3xlog$_{10}$TCNE | Int. | 0.100 | 0.627 | 0.315 | -0.13–0.785 |
| MDxM1xA | Int. | 0.100 | 0.298 | -0.028 | -0.236–0.173 |
| MDxM2xA | Int. | 0.100 | 0.298 | 0.031 | -0.173–0.241 |
| MDxM3xA | Int. | 0.100 | 0.298 | -0.023 | -0.238–0.196 |
| MDxM1xPTA$_{1-2}$ | Int. | 0.100 | 0.087 | 0.001 | -0.057–0.064 |
| MDxM2xPTA$_{1-2}$ | Int. | 0.100 | 0.087 | -0.005 | -0.066–0.056 |
| MDxM3xPTA$_{1-2}$ | Int. | 0.100 | 0.087 | -0.001 | -0.067–0.067 |
| MDxM1xlog$_{10}$TCNE | Int. | 0.100 | 0.627 | 0.039 | -0.37–0.455 |
| MDxM2xlog$_{10}$TCNE | Int. | 0.100 | 0.627 | -0.010 | -0.413–0.406 |
| MDxM3xlog$_{10}$TCNE | Int. | 0.100 | 0.627 | -0.071 | -0.505–0.379 |
| CFxMDxA | Int. | 0.100 | 0.298 | -0.035 | -0.16–0.093 |
| CFxMDxPTA$_{1-2}$ | Int. | 0.100 | 0.087 | 0.003 | -0.036–0.041 |
| CFxMDxlog$_{10}$TCNE | Int. | 0.100 | 0.627 | 0.086 | -0.173–0.334 |
| CFxMDxM1xA | Int. | 0.100 | 0.298 | -0.017 | -0.225–0.187 |
| CFxMDxM1xPTA$_{1-2}$ | Int. | 0.100 | 0.087 | 0.002 | -0.06–0.064 |
| CFxMDxM1xlog$_{10}$TCNE | Int. | 0.100 | 0.627 | 0.007 | -0.396–0.419 |
| CFxMDxM2xA | Int. | 0.100 | 0.298 | 0.010 | -0.196–0.214 |
| CFxMDxM2xPTA$_{1-2}$ | Int. | 0.100 | 0.087 | -0.000 | -0.063–0.061 |
| CFxMDxM2xlog$_{10}$TCNE | Int. | 0.100 | 0.627 | 0.026 | -0.38–0.434 |
| CFxMDxM3xA | Int. | 0.100 | 0.298 | 0.000 | -0.22–0.213 |
| CFxMDxM3xPTA$_{1-2}$ | Int. | 0.100 | 0.087 | 0.002 | -0.066–0.069 |
| CFxMDxM3xlog$_{10}$TCNE | Int. | 0.100 | 0.627 | -0.040 | -0.478–0.389 |

Table S8: Model terms and coefficients for the FFR TFS SNR model.

| Coefficient | Type | Prior scale $z$ | Prior scale | Posterior Median | 99% CI |
|---|---|---|---|---|---|
| Intercept | | 2.000 | 7.632 | 5.300 | 5.133–5.464 |
| A | Cont. | 0.100 | 0.216 | -0.588 | -1.123– -0.041 |
| $PTA_{1-2}$ | Cont. | 0.100 | 0.063 | 0.002 | -0.122–0.13 |
| $\log_{10}TCNE$ | Cont. | 0.100 | 0.455 | -0.508 | -1.562–0.356 |
| $\sqrt[3]{Y}$ | Cont. | 0.100 | 0.336 | 0.283 | -0.338–1.027 |
| M1 | Cat. | 10.000 | 38.159 | -0.392 | -0.692– -0.106 |
| M2 | Cat. | 10.000 | 38.159 | 0.257 | -0.01–0.524 |
| M3 | Cat. | 10.000 | 38.159 | -0.498 | -0.79– -0.213 |
| M1xA | Int. | 0.100 | 0.216 | 0.178 | 0.013–0.351 |
| $M1xPTA_{1-2}$ | Int. | 0.100 | 0.063 | 0.039 | -0.008–0.088 |
| $M1x\log_{10}TCNE$ | Int. | 0.100 | 0.455 | 0.129 | -0.176–0.447 |
| M2xA | Int. | 0.100 | 0.216 | -0.038 | -0.188–0.116 |
| $M2xPTA_{1-2}$ | Int. | 0.100 | 0.063 | -0.019 | -0.063–0.026 |
| $M2x\log_{10}TCNE$ | Int. | 0.100 | 0.455 | -0.035 | -0.331–0.256 |
| M3xA | Int. | 0.100 | 0.216 | 0.020 | -0.143–0.183 |
| $M3xPTA_{1-2}$ | Int. | 0.100 | 0.063 | 0.007 | -0.041–0.056 |
| $M3x\log_{10}TCNE$ | Int. | 0.100 | 0.455 | 0.013 | -0.315–0.331 |

Table S9: Model terms and coefficients for the FFR ENV latency model.

| Coefficient | Type | Prior scale $z$ | Prior scale | Posterior Median | 99% CI |
|---|---|---|---|---|---|
| Intercept | | 2.000 | 5.581 | 13.951 | 13.803–14.101 |
| A | Cont. | 0.100 | 0.158 | -0.077 | -0.39–0.189 |
| $PTA_{1-2}$ | Cont. | 0.100 | 0.046 | 0.017 | -0.059–0.106 |
| $\log_{10}TCNE$ | Cont. | 0.100 | 0.333 | 0.099 | -0.456–0.684 |
| $\sqrt[3]{Y}$ | Cont. | 0.100 | 0.246 | 0.050 | -0.349–0.477 |
| CF | Cat. | 10.000 | 27.907 | -0.318 | -0.455– -0.185 |
| MD | Cat. | 10.000 | 27.907 | -0.089 | -0.209–0.032 |
| M1 | Cat. | 10.000 | 27.907 | -0.727 | -0.92– -0.529 |
| M2 | Cat. | 10.000 | 27.907 | 0.136 | -0.063–0.338 |
| M3 | Cat. | 10.000 | 27.907 | 0.041 | -0.183–0.258 |
| CFxMD | Int. | 0.100 | 0.279 | -0.098 | -0.211–0.019 |

| | | | | | |
|---|---|---|---|---|---|
| CFxM1 | Int. | 0.100 | 0.279 | -0.100 | -0.282–0.089 |
| CFxM2 | Int. | 0.100 | 0.279 | 0.062 | -0.121–0.255 |
| CFxM3 | Int. | 0.100 | 0.279 | 0.083 | -0.12–0.288 |
| CFxA | Int. | 0.100 | 0.158 | -0.109 | -0.187– -0.033 |
| CFxPTA$_{1-2}$ | Int. | 0.100 | 0.046 | -0.003 | -0.028–0.021 |
| CFxlog$_{10}$TCNE | Int. | 0.100 | 0.333 | 0.065 | -0.089–0.22 |
| MDxM1 | Int. | 0.100 | 0.279 | -0.009 | -0.186–0.177 |
| MDxM2 | Int. | 0.100 | 0.279 | 0.013 | -0.168–0.203 |
| MDxM3 | Int. | 0.100 | 0.279 | 0.022 | -0.173–0.222 |
| MDxA | Int. | 0.100 | 0.158 | 0.025 | -0.045–0.093 |
| MDxPTA$_{1-2}$ | Int. | 0.100 | 0.046 | 0.011 | -0.01–0.033 |
| MDxlog$_{10}$TCNE | Int. | 0.100 | 0.333 | -0.125 | -0.267–0.01 |
| M1xA | Int. | 0.100 | 0.158 | -0.041 | -0.149–0.064 |
| M1xPTA$_{1-2}$ | Int. | 0.100 | 0.046 | -0.014 | -0.048–0.02 |
| M1xlog$_{10}$TCNE | Int. | 0.100 | 0.333 | 0.060 | -0.157–0.282 |
| M2xA | Int. | 0.100 | 0.158 | 0.006 | -0.099–0.118 |
| M2xPTA$_{1-2}$ | Int. | 0.100 | 0.046 | 0.014 | -0.019–0.05 |
| M2xlog$_{10}$TCNE | Int. | 0.100 | 0.333 | -0.006 | -0.231–0.216 |
| M3xA | Int. | 0.100 | 0.158 | -0.015 | -0.133–0.099 |
| M3xPTA$_{1-2}$ | Int. | 0.100 | 0.046 | -0.016 | -0.055–0.021 |
| M3xlog$_{10}$TCNE | Int. | 0.100 | 0.333 | -0.145 | -0.387–0.091 |
| CFxM1xA | Int. | 0.100 | 0.158 | 0.010 | -0.095–0.116 |
| CFxM2xA | Int. | 0.100 | 0.158 | 0.021 | -0.084–0.132 |
| CFxM3xA | Int. | 0.100 | 0.158 | -0.037 | -0.152–0.08 |
| CFxM1xPTA$_{1-2}$ | Int. | 0.100 | 0.046 | -0.005 | -0.039–0.028 |
| CFxM2xPTA$_{1-2}$ | Int. | 0.100 | 0.046 | 0.002 | -0.032–0.037 |
| CFxM3xPTA$_{1-2}$ | Int. | 0.100 | 0.046 | -0.003 | -0.04–0.035 |
| CFxM1xlog$_{10}$TCNE | Int. | 0.100 | 0.333 | 0.020 | -0.197–0.236 |
| CFxM2xlog$_{10}$TCNE | Int. | 0.100 | 0.333 | 0.058 | -0.161–0.283 |
| CFxM3xlog$_{10}$TCNE | Int. | 0.100 | 0.333 | -0.135 | -0.383–0.095 |
| MDxM1xA | Int. | 0.100 | 0.158 | 0.027 | -0.076–0.132 |
| MDxM2xA | Int. | 0.100 | 0.158 | -0.019 | -0.124–0.09 |
| MDxM3xA | Int. | 0.100 | 0.158 | -0.004 | -0.118–0.107 |
| MDxM1xPTA$_{1-2}$ | Int. | 0.100 | 0.046 | -0.012 | -0.045–0.02 |
| MDxM2xPTA$_{1-2}$ | Int. | 0.100 | 0.046 | 0.006 | -0.027–0.04 |
| MDxM3xPTA$_{1-2}$ | Int. | 0.100 | 0.046 | -0.002 | -0.039–0.034 |
| MDxM1xlog$_{10}$TCNE | Int. | 0.100 | 0.333 | 0.049 | -0.163–0.263 |
| MDxM2xlog$_{10}$TCNE | Int. | 0.100 | 0.333 | -0.001 | -0.215–0.216 |

| | | | | | |
|---|---|---|---|---|---|
| MDxM3x$\log_{10}$TCNE | Int. | 0.100 | 0.333 | 0.005 | -0.232–0.236 |
| CFxMDxA | Int. | 0.100 | 0.158 | -0.019 | -0.087–0.048 |
| CFxMDxPTA$_{1-2}$ | Int. | 0.100 | 0.046 | 0.003 | -0.018–0.025 |
| CFxMDx$\log_{10}$TCNE | Int. | 0.100 | 0.333 | -0.028 | -0.169–0.107 |
| CFxMDxM1xA | Int. | 0.100 | 0.158 | -0.039 | -0.144–0.067 |
| CFxMDxM1xPTA$_{1-2}$ | Int. | 0.100 | 0.046 | 0.006 | -0.027–0.039 |
| CFxMDxM1x$\log_{10}$TCNE | Int. | 0.100 | 0.333 | 0.040 | -0.173–0.254 |
| CFxMDxM2xA | Int. | 0.100 | 0.158 | -0.031 | -0.136–0.082 |
| CFxMDxM2xPTA$_{1-2}$ | Int. | 0.100 | 0.046 | 0.007 | -0.026–0.042 |
| CFxMDxM2x$\log_{10}$TCNE | Int. | 0.100 | 0.333 | 0.002 | -0.218–0.219 |
| CFxMDxM3xA | Int. | 0.100 | 0.158 | 0.054 | -0.061–0.168 |
| CFxMDxM3xPTA$_{1-2}$ | Int. | 0.100 | 0.046 | -0.005 | -0.042–0.03 |
| CFxMDxM3x$\log_{10}$TCNE | Int. | 0.100 | 0.333 | -0.071 | -0.299–0.168 |

# 4 Supplementary results

## 4.1 ABR wave amplitudes for the HF-IERL montage

ABR grand averages with the HF-IERL montage are shown in Fig. S15 separately for each age group. Fig. S16 shows the ABR wave I and V amplitudes measured for each participant in each condition as a function of age with the HF-IERL montage. Overall, the pattern of results as a function of age was very similar to that observed for the HF-ITPR montage.

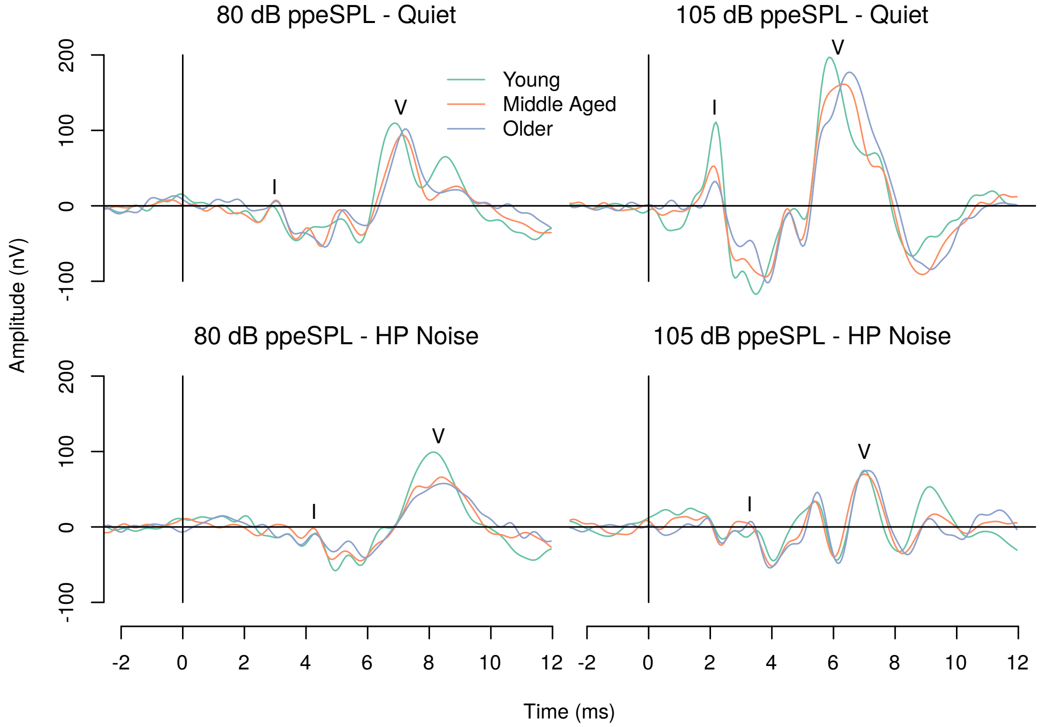


Figure S15: ABR grand averages with the HF-IERL montage for each experimental condition as a function of age group. It should be noted that although the grand averages are shown as a function of age group for illustration purposes here, the statistical analyses used age as a continuous rather than nominal variable.

The effects of age (Fig. S17), $PTA_{0.5-2}$ (Fig. S18), $PTA_{4-12}$ (Fig. S19), and $\log_{10}TCNE$ (Fig. S20) estimated by the Bayesian multiple regression models for

the HF-IERL montage were qualitatively similar to those described in the main manuscript for the HF-ITPR montage.

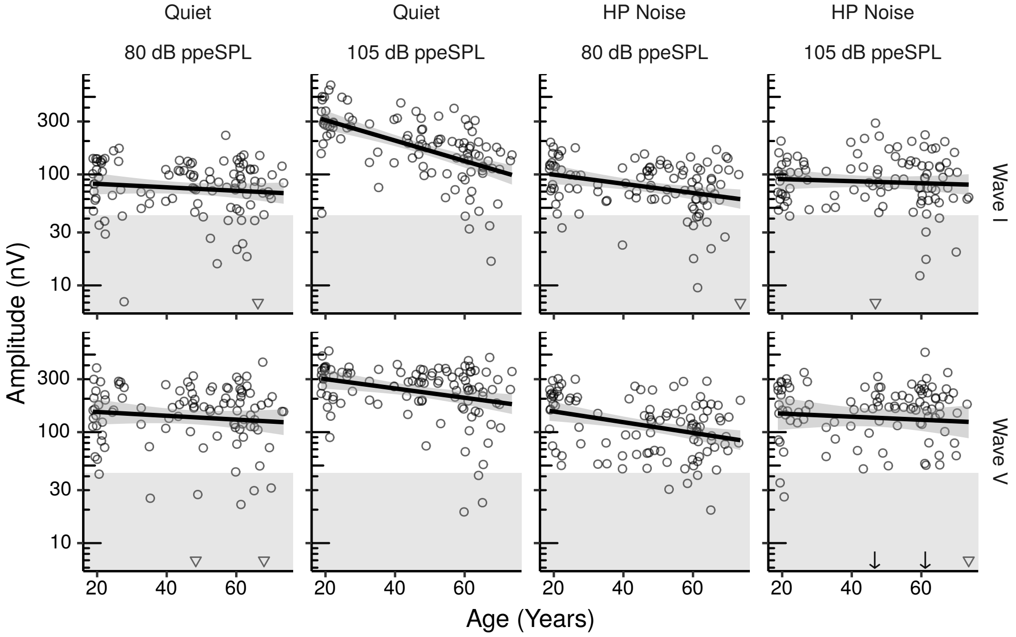


Figure S16: ABR wave I and V amplitudes by age for the HF-IERL montage. The inverted triangles represent data points for which the peak-trough amplitude could not be measured. These data points were modeled as having an amplitude lower than the lowest recorded peak-trough amplitude in the dataset through a censored analysis. The two downward arrows in the panel for wave V in HP noise represent the data points of a 47 years old participant with a peak-trough amplitude of 0.38 nV, and a 61 years old participant with a peak-trough amplitude of 3.35 nV. These two datapoints are not plotted at their actual coordinate simply for aesthetic reasons to avoid excessively enlarging the panel. Each panel shows a least squares line fit of wave amplitude by age with 95% confidence intervals as a visual aid. The slope for the effect of age estimated by the Bayesian multiple regression model is not the same as that shown in the figure.

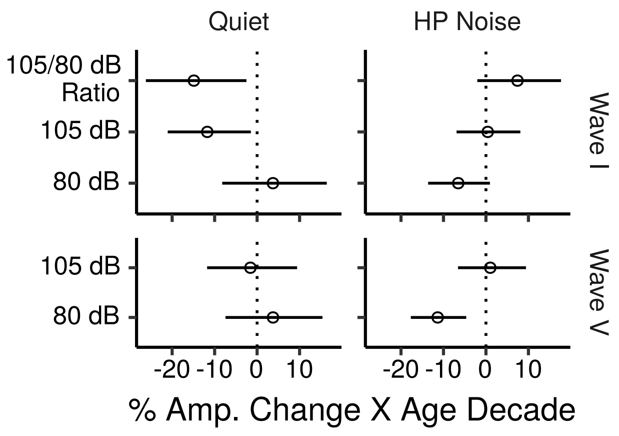


Figure S17: Posterior medians (circles) and 99% credibility intervals for the effects of age on ABR wave I and V amplitudes estimated by the Bayesian multiple regression models for the HF-IERL montage. Effects are plotted as percentage amplitude change for an age increase of 10 years.

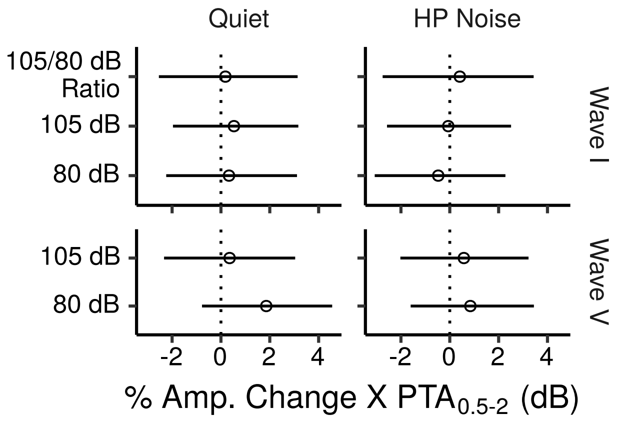


Figure S18: Posterior medians (circles) and 99% credibility intervals for the effects of $PTA_{0.5-2}$ on ABR wave I and V amplitudes estimated by the Bayesian multiple regression models for the HF-IERL montage.

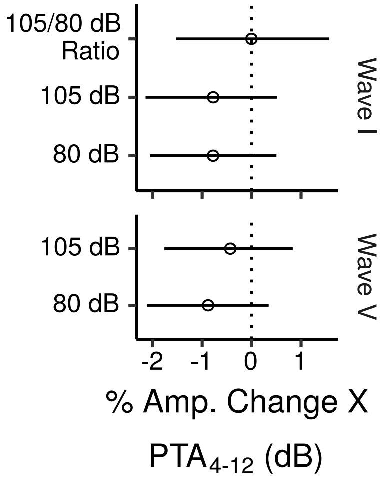


Figure S19: Posterior medians (circles) and 99% credibility intervals for the effects of $PTA_{4-12}$ on ABR wave I and V amplitudes estimated by the Bayesian multiple regression model for the HF-IERL montage.

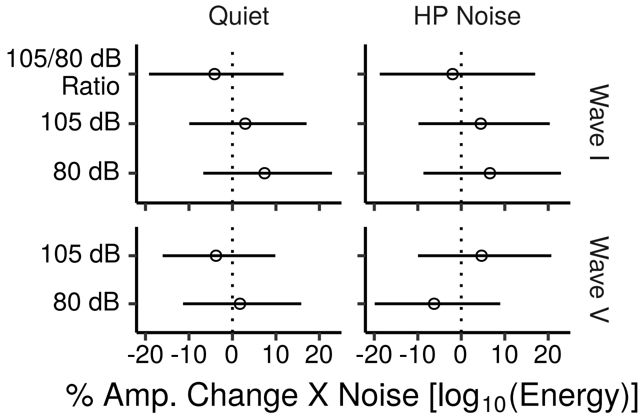


Figure S20: Posterior medians (circles) and 99% credibility intervals for the effects of $\log_{10}$TCNE on ABR wave I and V amplitudes estimated by the Bayesian multiple regression models for the HF-IERL montage.

## 4.2 ABR wave latencies for the HF-IERL montage

Fig. S21 shows the ABR wave latencies measured for each participant in each condition as a function of age for the HF-IERL montage. Qualitatively, the

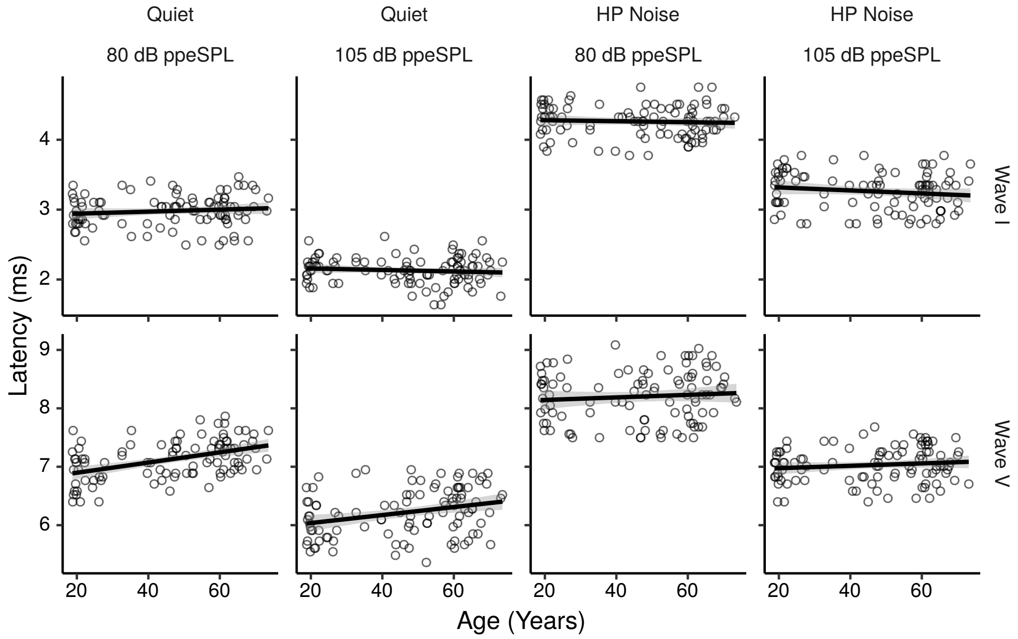


Figure S21: ABR wave I and V latencies by age for the HF-IERL montage. Each panel shows a least squares line fit of wave latency by age with 95% confidence intervals as a visual aid. The slope for the effect of age estimated by the Bayesian multiple regression model is not the same as that shown in the figure.

effects of age (Fig. S22), $PTA_{0.5-2}$ (Fig. S23), $PTA_{4-12}$ (Fig. S24), and $\log_{10}$TCNE (Fig. S25) estimated by the Bayesian multiple regression models for the HF-IERL montage were largely similar to those described in the main manuscript for the HF-ITPR montage.

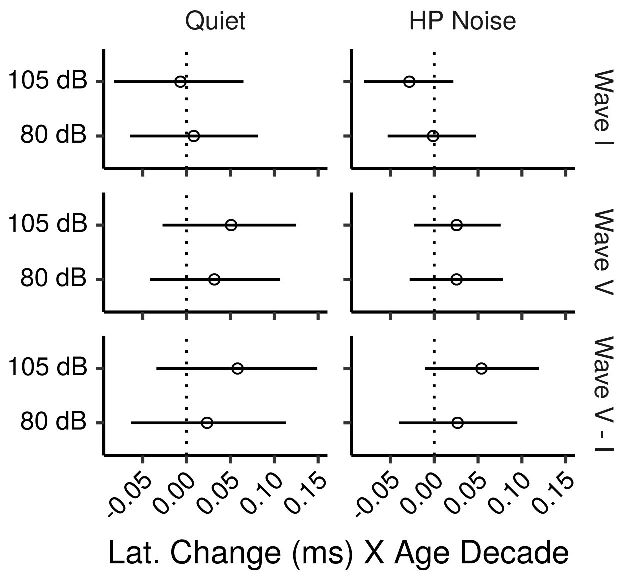


Figure S22: Posterior medians (circles) and 99% credibility intervals for the effects of age on ABR wave I and V latencies estimated by the Bayesian multiple regression models for the HF-IERL montage. The bottom row shows the effects for the wave I–V interpeak latencies. Effects are plotted as latency change for an age increase of 10 years.

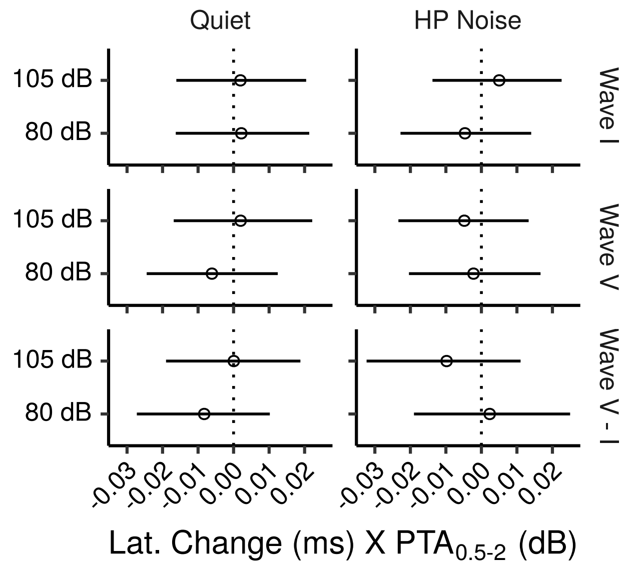


Figure S23: Posterior medians (circles) and 99% credibility intervals for the effects of $PTA_{0.5-2}$ on ABR wave I and V latencies estimated by the Bayesian multiple regression models for the HF-IERL montage. The bottom row shows the effects for the wave I–V interpeak latencies.

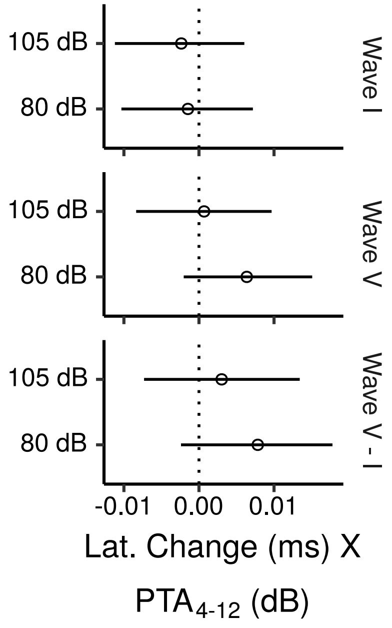


Figure S24: Posterior medians (circles) and 99% credibility intervals for the effects of $PTA_{4-12}$ on ABR wave I and V latencies estimated by the Bayesian multiple regression model for the HF-IERL montage. The bottom row shows the effects for the wave I–V interpeak latencies.

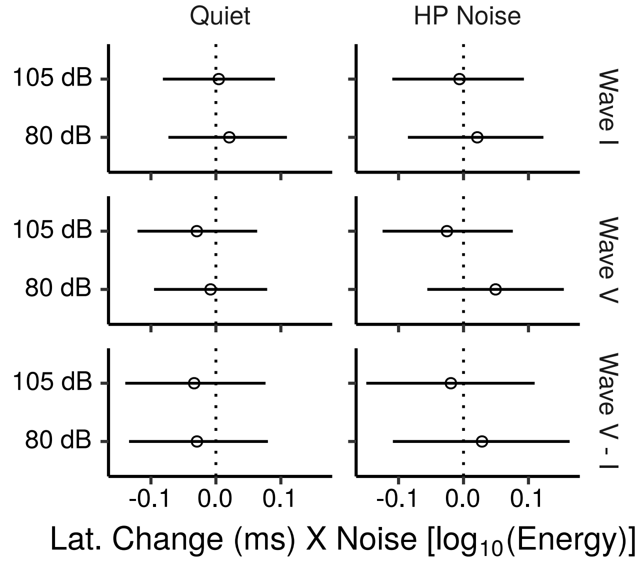


Figure S25: Posterior medians (circles) and 99% credibility intervals for the effects of $\log_{10}$TCNE on ABR wave I and V latencies estimated by the Bayesian multiple regression models for the HF-IERL montage. The bottom row shows the effects for the wave I–V interpeak latencies.

## 4.3 FFR ENV SNR results for each montage

Figs. S26, S27, S28, and S29 show the FFR ENV SNR measured for each participant in each condition as a function of age. Each of these figures shows the results for a different montage. The results for the HF-C7, HF-LERL, and HF-LTPR montages showed the same pattern described for the across-montage average in the main manuscript, while for the HF-LMST montage, SNRs tended to decrease with age not only for the 0.6-kHz CF, but also for the 2-kHz CF.

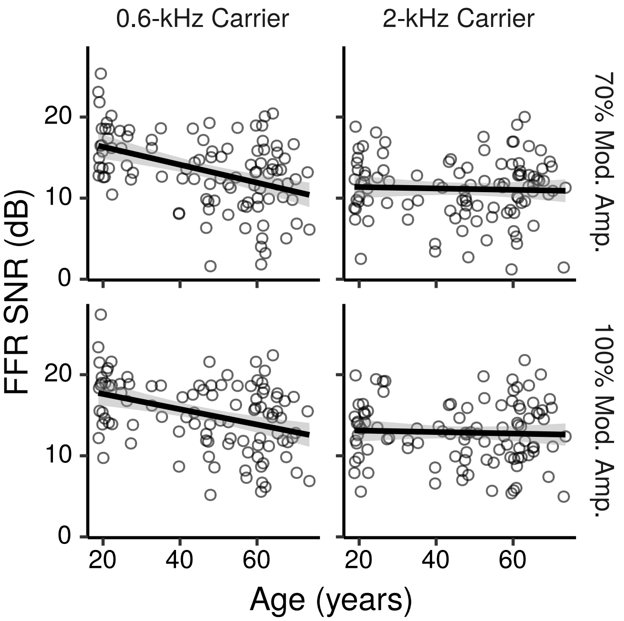


Figure S26: FFR ENV SNR by age for the HF-C7 montage. Each panel shows a least squares line fit of FFR SNR by age with 95% confidence intervals as a visual aid. The slope for the effect of age estimated by the Bayesian multiple regression model is not the same as that shown in the figure.

We chose SNR over raw signal level as a measure of FFR amplitude because the former measure is normalized, and easily interpretable in absolute terms. Nevertheless, i) scatterplots of raw signal levels by age showed essentially the same trends as the scatterplots of FFR SNR by age shown in Figs. S26, S27, S28, and S29, and ii) correlations between SNRs and raw signal levels were high ($\rho$ ranging from 0.68 to 0.88 for different montages); this suggests that

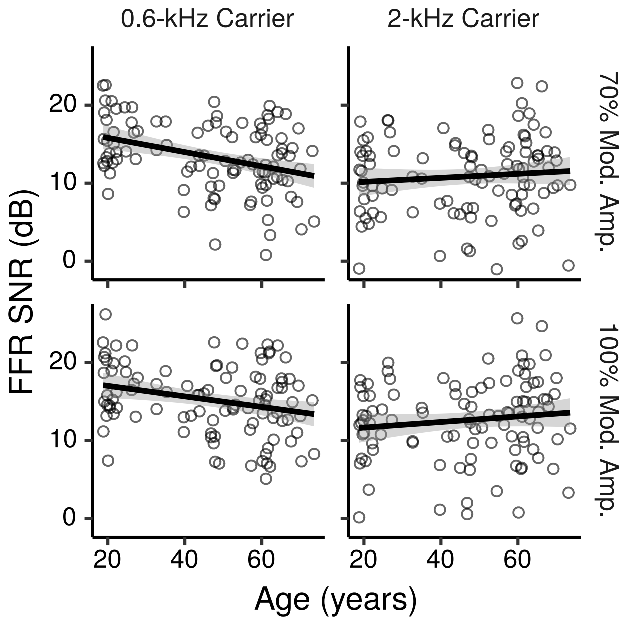


Figure S27: FFR ENV SNR by age for the HF-LERL montage. Each panel shows a least squares line fit of FFR SNR by age with 95% confidence intervals as a visual aid. The slope for the effect of age estimated by the Bayesian multiple regression model is not the same as that shown in the figure.

the effects of age on SNR were largely driven by age-related changes in signal levels rather than by age-related changes in noise levels.

Fig. S30 shows the CIs for the effects of age on FFR ENV SNR for each montage, as well as for the main effects across montages. The montage-specific effects were qualitatively similar to the main across-montage effects at the 0.6 kHz CF. At the 2-kHz CF, the effects for the HF-C7, HF-LERL, and HF-LTPR montages were also similar to the main across-montage effects, while for the HF-LMST montage there were trends for age-related decreases at both MDs (posterior median $\sim$ 0.6 dB per age decade).

Fig. S31 shows the CIs for the effects of $PTA_{1-2}$ on FFR ENV SNR for each montage, as well as for the main effects across montages. The montage-specific effects were qualitatively similar to the main across-montage effects at the 0.6 kHz CF. At the 2-kHz CF, the effects for the HF-C7, HF-LERL, and HF-LTPR montages were also similar to the main across-montage effects, with trends for

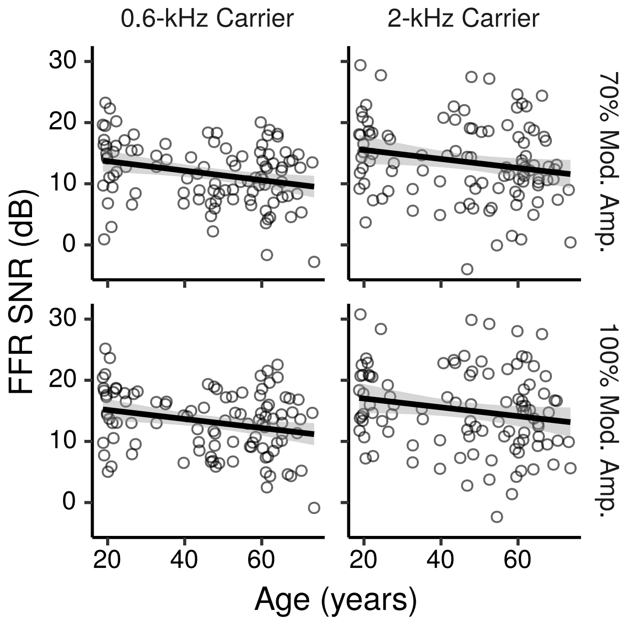


Figure S28: FFR ENV SNR by age for the HF-LMST montage. Each panel shows a least squares line fit of FFR SNR by age with 95% confidence intervals as a visual aid. The slope for the effect of age estimated by the Bayesian multiple regression model is not the same as that shown in the figure.

SNR increases with increasing $PTA_{1-2}$, while for the HF-LMST montage there were no trends for SNR increases with increasing $PTA_{1-2}$.

The effects of $\log_{10}$TCNE for each montage are shown in Fig. S32, and overall, are qualitatively similar to the main effects across montages described in the main manuscript.

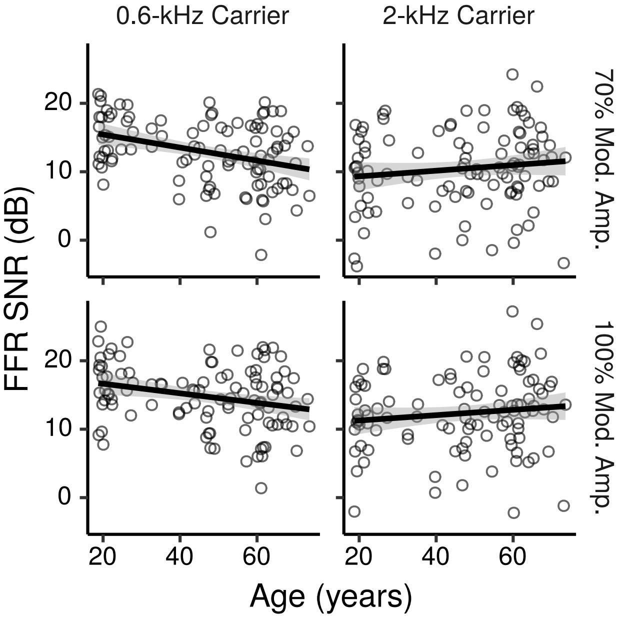


Figure S29: FFR ENV SNR by age for the HF-LTPR montage. Each panel shows a least squares line fit of FFR SNR by age with 95% confidence intervals as a visual aid. The slope for the effect of age estimated by the Bayesian multiple regression model is not the same as that shown in the figure.

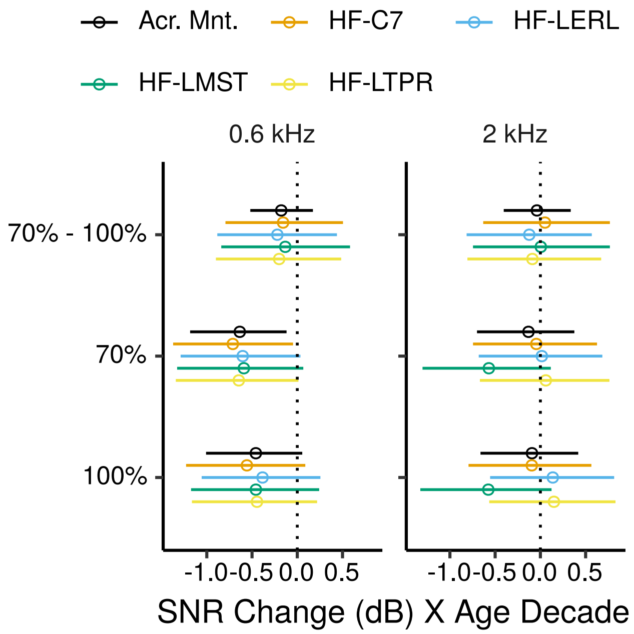


Figure S30: Posterior medians (circles) and 99% credibility intervals for the effects of age on FFR ENV SNR estimated by the Bayesian multiple regression model. The black "Acr. Mnt." segments plot the main effects across all montages. The top row shows the effect difference between the 70% and 100% MD. Effects are plotted as an SNR change for an age increase of ten years.

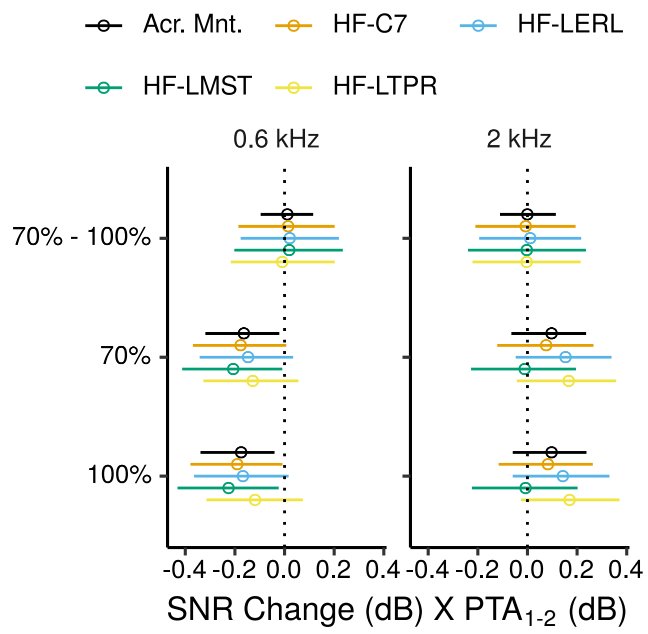


Figure S31: Posterior medians (circles) and 99% credibility intervals for the effects of $PTA_{1-2}$ on FFR ENV SNR estimated by the Bayesian multiple regression model. The black "Acr. Mnt." segments plot the main effects across all montages. The top row shows the effect difference between the 70% and 100% MD.

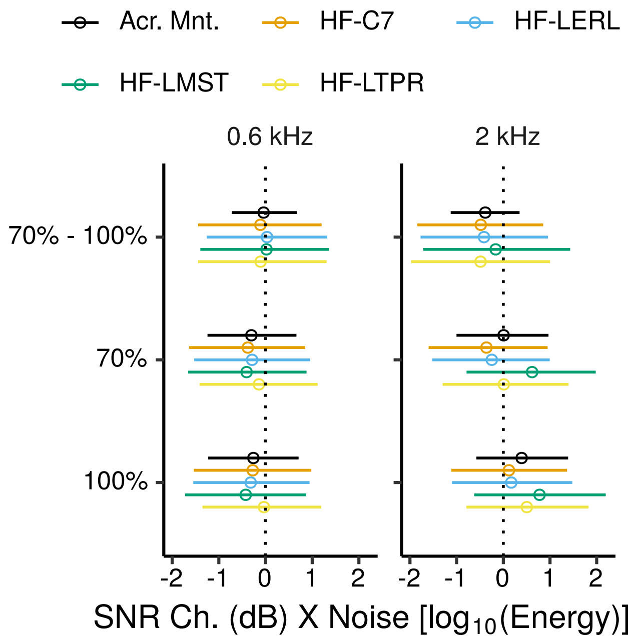


Figure S32: Posterior medians (circles) and 99% credibility intervals for the effects of $\log_{10}$TCNE on FFR ENV SNR estimated by the Bayesian multiple regression model. The black "Acr. Mnt." segments plot the main effects across all montages. The top row shows the effect difference between the 70% and 100% MD.

## 4.4 FFR TFS SNR results for each montage

Fig. S33 shows, for each montage, the FFR TFS SNR measured for each participant in each condition as a function of age. Overall, the montage-specific effects of age (Fig. S34), $PTA_{1-2}$ (Fig. S35), and $\log_{10}$TCNE (Fig. S36) were qualitatively similar to the main across-montage effects of these predictors described in the main manuscript.

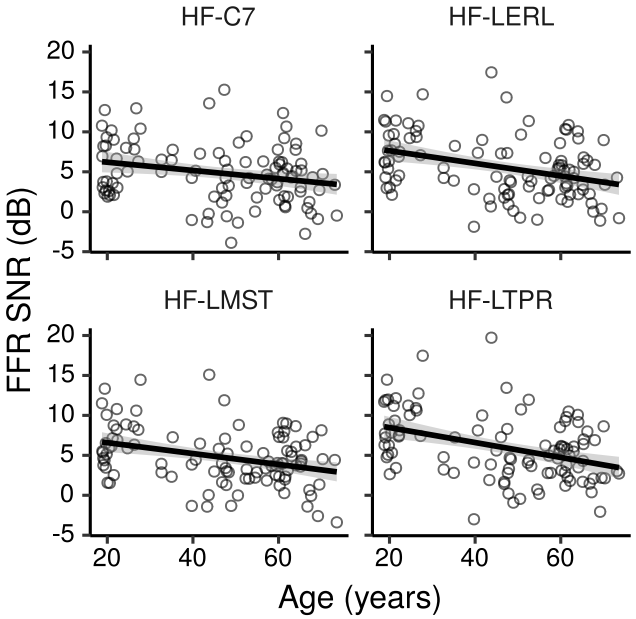


Figure S33: FFR TFS SNR by age for each electrode montage. For each panel the figure shows a least squares line fit of FFR SNR by age with 95% confidence intervals as a visual aid. The slope for the effect of age estimated by the Bayesian multiple regression model is not the same as that shown in the figure.

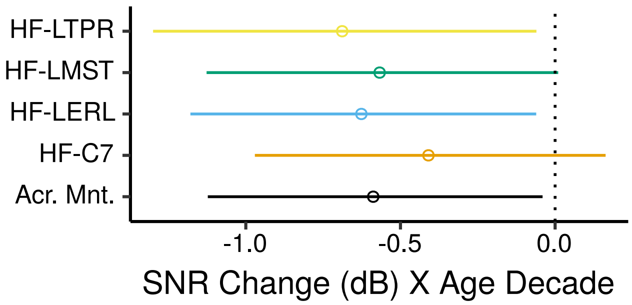


Figure S34: Posterior medians (circles) and 99% credibility intervals for the effects of age on FFR TFS SNR estimated by the Bayesian multiple regression model. The black “Acr. Mnt.” segment plots the main effect across all montages. Effects are plotted as an SNR change for an age increase of ten years.

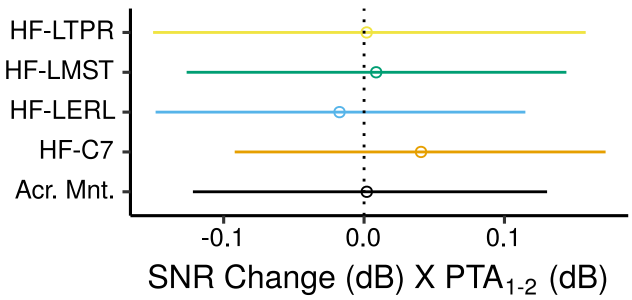


Figure S35: Posterior medians (circles) and 99% credibility intervals for the effects of $PTA_{1-2}$ on FFR TFS SNR estimated by the Bayesian multiple regression model. The black “Acr. Mnt.” segment plots the main effect across all montages.

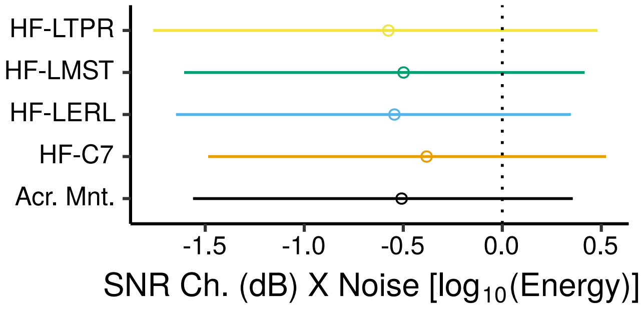


Figure S36: Posterior medians (circles) and 99% credibility intervals for the effects of $\log_{10}$TCNE on FFR TFS SNR estimated by the Bayesian multiple regression model. The black "Acr. Mnt." segment plots the main effect across all montages.

## 4.5 FFR ENV latency results for each montage

Figs. S37, S38, S39, and S40 show the FFR ENV latency measured for each participant in each condition as a function of age. Each of these figures shows the results for a different montage. The results of the Bayesian model indicate the presence of systematic latency differences across montages, with shorter than average latencies for the HF-C7 montage, longer latencies for the HF-ITPR montage, and a trend for slightly longer latencies for the HF-LERL montage [posterior median differences from the across-montage average, in ms: HF-C7 = -0.73 (CI: -0.92– -0.53); HF-LERL = 0.14 (CI:-0.06 – 0.34); HF-LMST = 0.04 (CI: -0.18 – 0.26); HF-LTPR = 0.55 (CI: 0.34 – 0.76)]. Overall, the montage-specific effects of age (Fig. S41), $PTA_{1-2}$ (Fig. S42), and $\log_{10}$TCNE (Fig. S43) were qualitatively similar to the main across-montage effects of these predictors described in the main manuscript.

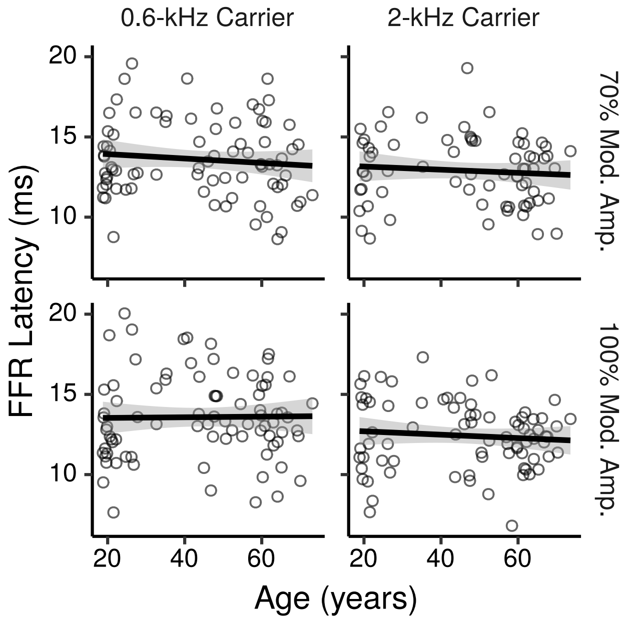


Figure S37: FFR ENV latency by age for the HF-C7 montage. Each panel shows a least squares line fit of FFR latency by age with 95% confidence intervals as a visual aid. The slope for the effect of age estimated by the Bayesian multiple regression model is not the same as that shown in the figure.

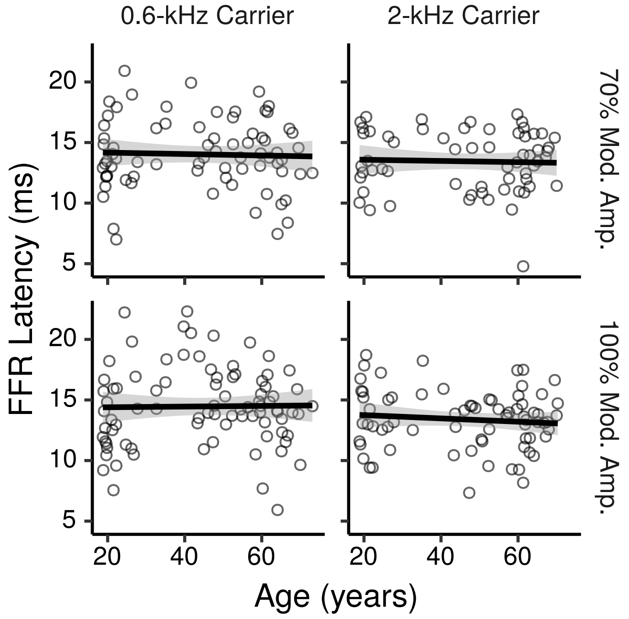


Figure S38: FFR ENV latency by age for the HF-LERL montage. Each panel shows a least squares line fit of FFR latency by age with 95% confidence intervals as a visual aid. The slope for the effect of age estimated by the Bayesian multiple regression model is not the same as that shown in the figure.

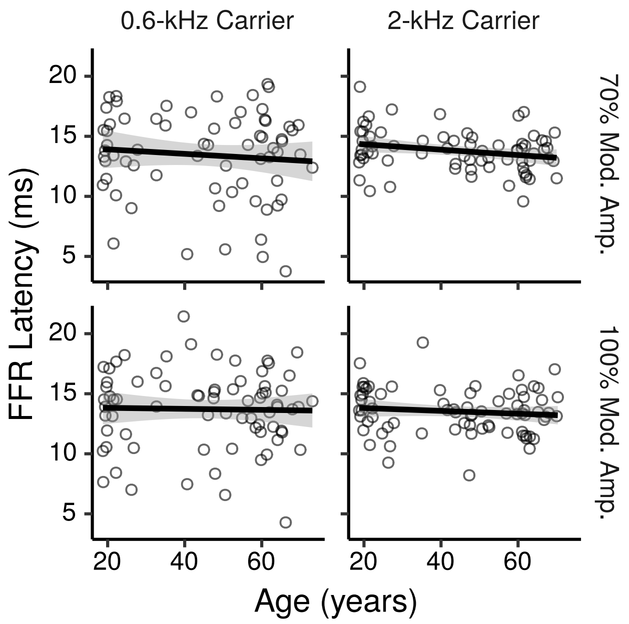


Figure S39: FFR ENV latency by age for the HF-LMST montage. Each panel shows a least squares line fit of FFR latency by age with 95% confidence intervals as a visual aid. The slope for the effect of age estimated by the Bayesian multiple regression model is not the same as that shown in the figure.

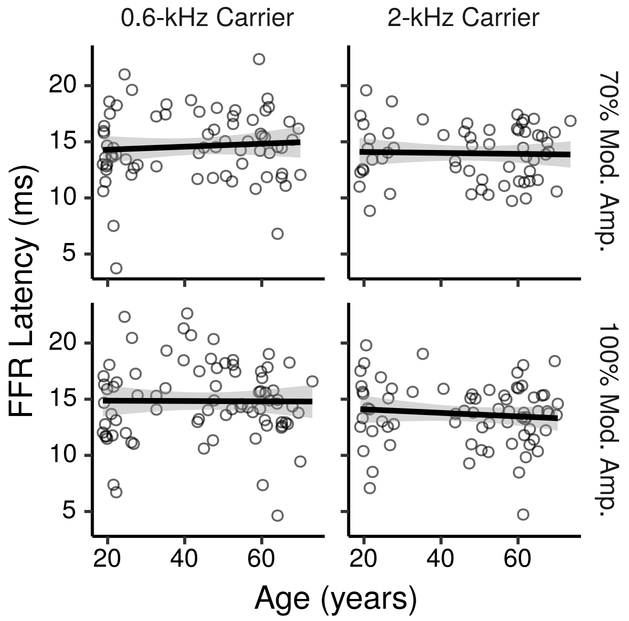


Figure S40: FFR ENV latency by age for the HF-LTPR montage. Each panel shows a least squares line fit of FFR latency by age with 95% confidence intervals as a visual aid. The slope for the effect of age estimated by the Bayesian multiple regression model is not the same as that shown in the figure.

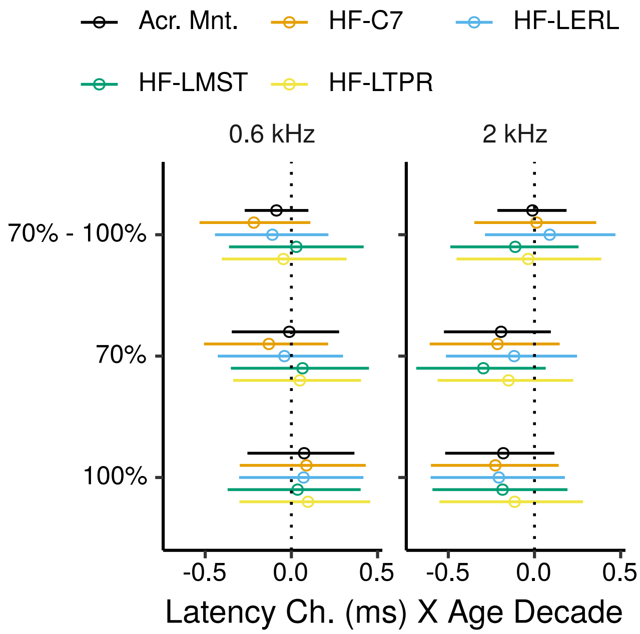


Figure S41: Posterior medians (circles) and 99% credibility intervals for the effects of age on FFR ENV latency estimated by the Bayesian multiple regression model. The black "Acr. Mnt." segments plot the main effects across all montages. The top row shows the effect difference between the 70% and 100% MD. Effects are plotted as a latency change, in ms, for an age increase of ten years.

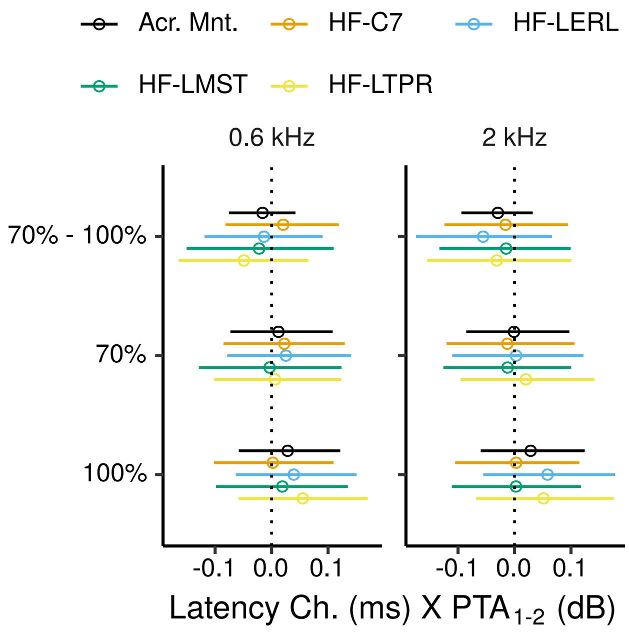


Figure S42: Posterior medians (circles) and 99% credibility intervals for the effects of $PTA_{1-2}$ on FFR ENV latency estimated by the Bayesian multiple regression model. The black "Acr. Mnt." segments plot the main effects across all montages. The top row shows the effect difference between the 70% and 100% MD.

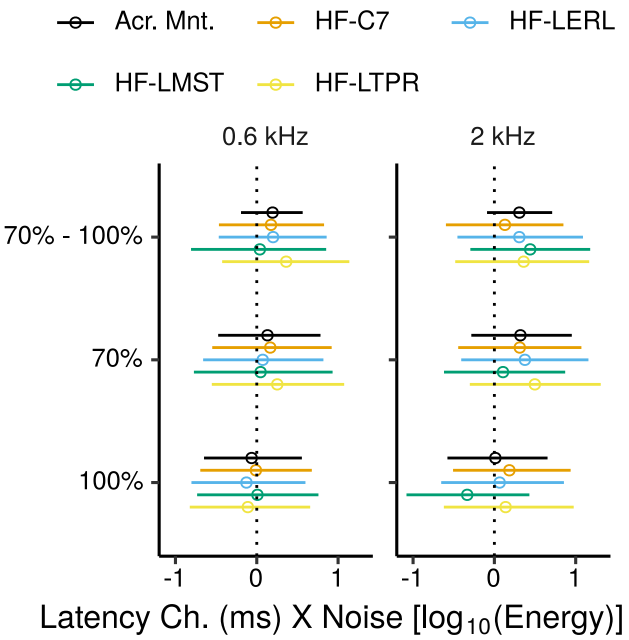


Figure S43: Posterior medians (circles) and 99% credibility intervals for the effects of $\log_{10}$TCNE on FFR ENV latency estimated by the Bayesian multiple regression model. The black "Acr. Mnt." segments plot the main effects across all montages. The top row shows the effect difference between the 70% and 100% MD.

# References

G. Prendergast, H. Guest, K. J. Munro, K. Kluk, A. Leger, D. A. Hall, M. G. Heinz, and C. J. Plack. Effects of noise exposure on young adults with normal audiograms I: Electrophysiology. *Hear. Res.*, 344:68–81, 2017. doi: 10.1016/j.heares.2016.10.028.

C. D. Bauch and W. O. Olsen. Comparison of ABR amplitudes with TIPtrode and mastoid electrodes. *Ear Hear.*, 11(6):463—-467, 1990. doi: 10.1097/00003446-199012000-00010.

G. Prendergast, W. Tu, H. Guest, R. E. Millman, K. Kluk, S. Couth, K. J. Munro, and C. J. Plack. Supra-threshold auditory brainstem response amplitudes in humans: Test-retest reliability, electrode montage and noise exposure. *Hear. Res.*, 364:38–47, 2018. doi: 10.1016/j.heares.2018.04.002.

A. Gelman. Multilevel (hierarchical) modeling: What it can and cannot do. *Technometrics*, 48(3):432–435, 2006. doi: 10.1198/004017005000000661.

A. Gelman and J. Hill. *Data analysis using regression and multilevel/hierarchical models*. Cambridge University Press, Cambridge, 2007. ISBN 978-0-521-68689-1.

A. P. Bradley and W. J. Wilson. Automated analysis of the auditory brainstem response. In *Proceedings of the 2004 Intelligent Sensors, Sensor Networks and Information Processing Conference, 2004.*, pages 541–545, 2004. doi: 10.1109/ISSNIP.2004.1417519.

A. Issa and H. F. Ross. An improved procedure for assessing ABR latency in young subjects based on a new normative data set. *Int. J. Pediatr. Otorhinolaryngol.*, 32(1):35–47, 1995. doi: 10.1016/0165-5876(94)01110-J.

M. E. Lutman, A. C. Davis, and M. A. Ferguson. *Epidemiological evidence for the effectiveness of the noise at work regulations. Research report RR669*. Health and Safety Executive, 2008.

H. Levitt. Transformed up-down methods in psychoacoustics. *J. Acoust. Soc. Am.*, 49(2):467–477, 1971. doi: 10.1121/1.1912375.

M. Plummer. JAGS: A program for analysis of Bayesian graphical models using Gibbs sampling. In Kurt Hornik, Friedrich Leisch, and Achim Zeileis,

editors, *Proceedings of the 3rd International Workshop on Distributed Statistical Computing*, Vienna, Austria, 2003.

R Core Team. *R: A Language and Environment for Statistical Computing*. R Foundation for Statistical Computing, Vienna, Austria, 2020. URL https://www.R-project.org/.

M. D. Lee and E.-J. Wagenmakers. *Bayesian cognitive modeling: A practical course*. Cambridge University Press, 2014. doi: 10.1017/CBO9781139087759.

J. K. Kruschke. *Doing Bayesian data analysis, a tutorial with R, JAGS, and Stan*. Elsevier, London, $2^{nd}$ edition, 2014. ISBN 978-0-12-405916-0.

L. S. Aiken, S. G. West, and R. R. Reno. *Multiple regression: Testing and interpreting interactions*. SAGE Publications, London, UK, 1991. ISBN 9780761907121.

R. J. Friedrich. In defense of multiplicative terms in multiple regression equations. *Am. J. Pol. Sci.*, 26(4):797, 1982. doi: 10.2307/2110973.

M. Don and J. J. Eggermont. Analysis of the click-evoked brainstem potentials in man unsing high-pass noise masking. *J. Acoust. Soc. Am.*, 63(4):1084–1092, 1978. doi: 10.1121/1.381816.

J. J. Eggermont and M. Don. Analysis of the click-evoked brainstem potentials in humans using high-pass noise masking. II. Effect of click intensity. *J. Acoust. Soc. Am.*, 68(6):1671–1675, 1980. doi: 10.1121/1.385199.

M. Ezekiel. A method of handling curvilinear correlation for any number of variables. *J. Am. Stat. Assoc.*, 19(148):431–453, 1924. doi: 10.1080/01621459.1924.10502899.

S. Chatterjee and A. S. Hadi. *Regression analysis by example*. John Wiley & Sons, Inc., Hoboken, New Jersey, 4th edition, 2006. doi: 10.1198/tech.2007.s498.

J. Fox. *Applied regression analysis and generalized linear models*. SAGE Publications, Thousand Oaks, CA, third edition edition, 2016. ISBN 978-1-4833-2131-8.
